# Supplementary material for: Sex-specific associations between surgery-induced weight loss and cancer outcomes: A post hoc analysis of the prospective, controlled Swedish Obese Subjects study
Source: PLoS Med. 2026 Jan 5;23(1):e1004876. doi: 10.1371/journal.pmed.1004876 (PMC12768343; doi:10.1371/journal.pmed.1004876)
Supplement: S1 Appendix — Table A. Obesity-related and non-obesity-related cancer events by sex and treatment. Fig A. Flow diagram of patient recruitment in the SOS study. Fig B. Changes in body mass index over 20 years in control participants and patients undergoing surgery, stratified by sex. Fig C. Sensitivity analysis of overall cancer incidence in women with follow-up starting 3 years after baseline. Fig D. Sensitivity analysis of overall cancer incidence in women with follow-up starting 3 years after baseline; individuals with cancer events prior to baseline excluded. Fig E. Incidence of non-obesity-related and non-female-specific cancer incidence in women from the surgery and control groups. Fig F. Incidence of non-obesity-related, and non-female-specific cancer in women from the surgery and control groups, stratified by baseline insulin levels. Fig G. Incidence of overall, obesity-related, and female-specific cancer from start of year 4 and onwards, in women from the surgery group, stratified by 1-year weight loss. (DOCX) [file pmed.1004876.s002.docx]

## S1 Appendix

| **Table of contents** | **Page** |
| --- | --- |
| **Table A.** Obesity-related and non-obesity-related cancer events by sex and treatment | 2 |
| **Fig A.** Flow diagram of patient recruitment in the SOS study | 3 |
| **Fig B.** Changes in body mass index over 20 years in controls and surgery patients, stratified by sex | 4 |
| **Fig C.** Sensitivity analysis of overall cancer incidence in women with follow-up starting 3 years after baseline | 5 |
| **Fig D.** Sensitivity analysis of overall cancer incidence in women with follow-up starting 3 years after baseline; individuals with cancer events prior to baseline excluded | 6 |
| **Fig E.** Incidence of non-obesity-related and non-female-specific cancer incidence in women from the surgery and control groups | 7 |
| **Fig F.** Incidence of non-obesity-related, and non-female-specific cancer in women from the surgery and control groups, stratified by baseline insulin levels | 8 |
| **Fig G.** Incidence of overall, obesity-related and female-specific cancer from start of year 4 and onwards, in women from the surgery group, stratified by 1-year weight loss | 9 |

### **Table A.** Obesity-related and non-obesity-related cancer events by sex and treatment

|  | **Women** | | **Men** | |
| --- | --- | --- | --- | --- |
|  | **Surgery** | **Control** | **Surgery** | **Control** |
| **Obesity-related** | **214** | **276** | **60** | **57** |
| Female-specific^1^ | 108 | 174 | - | - |
| Upper digestive tract^2^ | 29 | 31 | 23 | 19 |
| Colorectal | 47 | 43 | 20 | 23 |
| Kidney | 14 | 10 | 11 | 10 |
| Other | 16 | 18 | 6 | 5 |
|  |  |  |  |  |
| **Non-obesity-related** | **159** | **165** | **116** | **101** |
| Female-specific^3^ | 34 | 31 | - | - |
| Male-specific | - | - | 42 | 38 |
| Hematopoietic | 17 | 37 | 11 | 9 |
| Skin | 50 | 47 | 22 | 29 |
| Respiratory | 19 | 17 | 13 | 9 |
| Other | 39 | 33 | 28 | 16 |

^1^ Postmenopausal breast, endometrium, ovary

^2^ Gallbladder, liver, pancreas, esophagus, stomach

^3^ Premenopausal breast, cervix, vulva, vagina

Note that the total number of cancer events reported here exceeds the number of incident cancers in Figure 1, as some individuals experienced multiple cancer events during follow-up (e.g., an obesity-related cancer and a non-obesity-related cancer). In the analysis of overall cancer incidence, only one, i.e. the first cancer event is accounted for to identify the first incident cancer. However, in the current analysis, individuals may have had more than one cancer event and therefore be accounted for more than one time.

### **Fig A.** Flow Diagram

### **Fig B.** Changes in body mass index over 20 years in control participants and patients undergoing surgery, stratified by sex

Lines are estimated means from a mixed model with adjustment for age. Dots represent observed values from individual participants.
The y axis is truncated at a body-mass index of 25 and of 55 kg/m^2^, but all observations were used in the estimation of means.

### **Fig C.** Sensitivity analysis of overall cancer incidence in women with follow-up starting 3 years after baseline

IR/1000, incidence rate per 1,000 person-years; 95% CI, 95% confidence interval; HR, Hazard Ratio; HRadj, HR adjusted for age, sagittal diameter, alcohol consumption, smoking, and serum insulin levels.

### **Fig D.** Sensitivity analysis of overall cancer incidence in women with follow-up starting 3 years after baseline; individuals with cancer events prior to baseline excluded

IR/1000, incidence rate per 1,000 person-years; 95% CI, 95% confidence interval; HR, Hazard Ratio; HRadj, HR adjusted for age, sagittal diameter, alcohol consumption, smoking, and serum insulin levels.

### **Fig E.** Cumulative incidence of non-obesity-related (A) and non-female-specific cancer (B) incidence in women from the surgery and control groups

IR/1000, incidence rate per 1,000 person-years; 95% CI, 95% confidence interval; HR, Hazard Ratio; HRadj, HR adjusted for age, sagittal diameter, alcohol consumption, smoking, and serum insulin levels.

**Fig F.** Cumulative incidence of non-obesity-related (A), and non-female-specific (B) cancer in women from the surgery and control groups, stratified by baseline insulin levels

IR/1000, incidence rate per 1,000 person-years; 95% CI, 95% confidence interval; HR, Hazard Ratio; HRadj, HR adjusted for age, sagittal diameter, alcohol consumption, smoking, and serum insulin levels.

**Fig G.** Unadjusted cumulative incidence of overall (A), obesity-related (B) and female-specific cancer (C) from start of year 4 and onwards, in women from the surgery group, stratified by 1-year weight loss

Cancer events that occurred during the first 3 years were excluded from the analyses. In women with missing information regarding bodyweight at 12 months, the 6-month weights were used. If both 6-month and 12-month weights were missing, median 1-year weight changes in women who underwent surgery were imputed.

IR/1000, incidence rate per 1,000 person-years; 95% CI, 95% confidence interval.
