# Supplementary material for: Sex-specific associations between surgery-induced weight loss and cancer outcomes: A post hoc analysis of the prospective, controlled Swedish Obese Subjects study
Source: PLoS Med. 2026 Jan 5;23(1):e1004876. doi: 10.1371/journal.pmed.1004876 (PMC12768343; doi:10.1371/journal.pmed.1004876)
Supplement: S2 Appendix — The SOS study was designed as a prospective, controlled intervention trial. The present analyses (insulin and cancer outcomes) were post hoc and were not prespecified in the original protocol. (PDF) [file pmed.1004876.s003.pdf]

This supplement contains the following items:

1. Original study protocol including power calculations and statistical description (in Swedish, from 1987)
2. English translation of rationale and aims from original study protocol
3. Ethical approval

Bil

FÖRSÖKSPROTOKOLL

SWEDISH OBESE SUBJECTS (SOS)

Registerstudie och prospektiv kontrollerad interventionsstudie av övervikt utnyttjande olika ventrikelingrepp som medel att uppnå viktnedgång och minskad recidivbenägenhet.

870113

INNEHÅLLSFÖRTECKNING


| | | Sida |
|---|---|---|
| SAMMANFATTNING | | 1 |
| FORSKNINGSPROGRAM | | 3 |
| 1 | MÅLSÄTTNING | 3 |
| 1.1 | Primära målsättningar | 3 |
| 1.2 | Sekundära målsättningar | 3 |
| 1.3 | Andra frågeställningar | 4 |
| 2 | BAKGRUND | 5 |
| 2.1 | Associationer mellan fetmagrad och risk | 5 |
| 2.2 | Associationer mellan fettvävsfördelning och risk | 5 |
| 2.3 | Kausalitet och riskreversibilitet | 5 |
| 2.4 | Tidigare behandlingsresultat | 6 |
| 2.4.1 | Icke-kirurgisk behandling | 6 |
| 2.4.2 | Kirurgisk behandling | 6 |
| 2.4.3 | Konklusion betr. tillgängliga behandlingsstudier | 7 |
| 3 | ARBETSPLAN | 8 |
| 3.1 | REGISTERSTUDIEN | 8 |
| 3.1.1 | Patienturval | 8 |
| 3.1.2 | Rekryteringsmetoder | 8 |
| 3.1.3 | Information frågeformulär och mätvariabler | 9 |
| 3.2 | INTERVENTIONSSTUDIEN | 10 |
| 3.2.1 | Rekrytering | 10 |
| 3.2.2 | Patienturval | 10 |
| 3.2.3 | Dimensionering och observationstid | 11 |
| 3.2.4 | Matchningsprocedur, inklusionsdatum och undersökningstillfällen | 11 |
| 3.2.5 | Behandling | 14 |
| 3.3 | REGISTRERING OCH VÄRDERING AV (EFFEKT-) VARIABLER OCH BIVERKNINGAR | 14 |
| 3.4 | STATISTIK | 15 |
| 3.5 | SPECIELLA ASPEKTER | 16 |
| 3.5.1 | Ekonomiska aspekter | 16 |
| 3.5.2 | Psykologiska aspekter | 16 |
| 3.5.3 | Genetiska aspekter | 16 |
| 4 | ETISKA ÖVERVÄGANDEN | 17 |
| 5 | SAMHÄLLSEKONOMISKA KONSEKVENSER | 18 |
| 6 | STUDIENS ADMINISTRATION | 18 |
| 7 | PILOTSTUDIE | 18 |
| 8 | REFERENSER | 20 |
| APPENDIX. ÄNDPUNKTSDEFINITIONER | | 24 |

SAMMANFATTNING

MÅLSÄTTNING

Studiens primära målsättning är att undersöka om den förhöjda mortaliteten vid fetma reduceras av viktnedgång.

Gastroplastikingrepp användes därvid som medel att uppnå långvarig viktnedgång. Bland sekundära målsättningar nämnes här försök att fastställa om viktreduktion minskar mortalitet och mobiditet i hjärt-kärlsjukdom, diabetes och gallvägssjukdom. Studien skall också belysa i vilken mån arv och miljö förklarar grad av fetma och viktnedgångsresultat. Psykologiska reaktioner av betydelse för terapisvikt samt förändringar i livskvalitet under behandling kommer att analyseras. Slutligen kommer effektivitetsmått att appliceras på kirurg- och kontrollgrupperna. Därvid ställes olika nyttovariabler (sänkt mortalitet och morbiditet, livskvalitet etc) i relation till individens och samhällets kostnader.

BAKGRUND

Ett U-format samband mellan kroppsvikt och mortalitet har påvisats i flera studier. I nyligen publicerade arbeten har vi visat att typ av fettvävsfördelning är en starkare prediktor för mortalitet och morbiditet än graden av total fetma. Acceptabla kontrollerade interventionsstudier vid fetma har aldrig utförts vad gäller kardiovaskulär sjuklighet och död. Fetmans kausala betydelse för riskökningen är därmed oklar och ev. riskreversibilitet vid viktnedgång obevisad. Alla icke-kirurgiska former av fetma är förenade med mycket dåliga långtidsresulat medan några typer av ventrikelingrepp ger god viktnedgång och en acceptabel biverkningsnivå.

ARBETSPLAN

Registerstudie. I en landsomfattande på distriktläkarmottagningarna utförd medicinsk undersökning insamlas information och mätvärden från 4000-10000 kraftigt överviktiga personer. Bland variablerna kan nämnas längd, vikt, omfångsmått, blodsocker, insulin, TG, cholesterol, HDL, elstatus, urat, leverstatus, EKG, blodtryck, fetmahereditet, psykosociala faktorer samt tidigare och aktuella sjukdomar. Registret utgör den huvudsakliga rekryteringskällan för interventionsstudien och är dessutom av värde som bakgrundssinformation t.ex. när man skall bedöma interventionsstudiens externa validitet.

Interventionsstudie. Patienter som i registerstudien förkarat sig intresserade av kirurgi kallas till kirurgpoliklinik för ytterligare information och undersökningar. Ett 20-tal svenska kirurgkliniker deltager i studien. För varje opererat kirurgfall tillföres ett eller - om registerstudiens storlek så tillåter - två kontrollfall. Inklusionsdag för kirurg- och kontrollfallen blir kirurgfallets operationsdag. Kontrollfallen väljes med en minimiseringsmetod så att kontroll- och kirurggruppernas medelvärden blir så lika som möjligt med avseende på 14 prognostiskt viktiga faktorer.

Kirurgfallen följs upp regelbundet vid vederbörande kirurgklinik och kontrollfallen inom primärvården. Studien skall pågå i 10 år och kommer att omfatta 2000 inkluderade kirurgfall och 2000-4000 kontrollfall.

Effektregistrering och analys. Studien administreras datamaskinellt. Mortalitet samt sjukskrivning och pensionering fastställes genom samkörning med Statistiska Centralbyråns resp. Försäkringskassans register. Kontrollgruppens viktförändringar och sjuklighet följes med enkäter samt 2 och 10-årsuppföljningar. Diagnos vid dödsfall och i samband med sjukhusvård inhämtas från dödsbevis resp. sjukhusjournaler. Vid mortalitetsjämförelser av kirurg- och kontrollgrupperna beaktas dödligheten från och med inklusionsdagen. Vid värdering av viktnedgångens betydelse bearbetas all mortalitet från och med dag 61 efter inklusionen.

Grad av viktöverensstämmelse bland biologiska och icke-biologiska släktingar över tre generationer analyseras med "path-analysis procedures" i samarbete med Laval-universitetet i Québec. Dessa metoder ger mått på familjär anhopning av fetma, sammanlevnadseffekter, transmissionseffekter mellan generation, maternella och paternella effekter samt uppskattningar av hereditära och miljömässiga inflytanden. Psykologiska variabler analyseras bl.a. med statistiska metoder för kausalanalys (LISREL-metoder).

## BETYDELSE

Studien har stor medicinsk, social och samhällsekonomisk betydelse eftersom mortalitet på grund av övervikt är av samma storleksordning (4%) som den på grund av lungcancer hos män eller bröstcancer hos kvinnor.

# FORSKNINGSPROGRAM

## SWEDISH OBESE SUBJECTS (SOS)

Registerstudie och prospektiv kontrollerad interventionsstudie av övervikt utnyttjande gastroplastik som ett medel att uppnå viktnedgång och minskad recidivbenägenhet.

## 1 MÅLSÄTTNING

SOS är dels en registerstudie av överviktiga, dels en prospektiv matchad interventionsstudie i vilken en kontrollgrupp omhändertages enligt rådande medicinska rutiner i primärvården och en grupp behandlas kirurgiskt med någon form av ventrikelingrepp på ett av ca 20 deltagande läns- eller regionssjukhus. Målsättningarna är:

### 1.1 Primära målsättningar:

1) att undersöka om viktreduktion minskar total mortalitet.

2) att undersöka om kirurgisk behandling minskar total mortalitet

### 1.2 Sekundära målsättningar

1) att undersöka om viktreduktion

   a) minskar mortalitet och morbiditet i hjärt-kärlsjukdom (hypertoni, angina pectoris, claudicatio intermittens, stroke och hjärtinfarkt), diabetes och gallvägssjukdom

   b) ger olika riskreversibilitet hos patienter med android och gynoid fettvävsfördelning

2) att undersöka om kirurgisk behandling

   a) minskar mortalitet och morbiditet i hjärt-kärlsjukdom (hypertoni, angina pectoris, claudicatio intermittens, stroke och hjärtinfarkt), diabetes och gallvägssjukdom

   b) ger olika riskreversibilitet hos patienter med android och gynoid fettvävsfördelning

3) psykologiska reaktioner undersökes genom

   a) att studera variationer i livskvalitet och kroppsuppfattning i kontroll- och kirurggrupp

   b) att finna psykologiska variabler av prediktivt värde för behandlingsresultat

   c) att studera viktrecidivets psykologi

4) att studera arvets, uppväxtmiljöns och den vuxna miljöns betydelse för grad av fetma och behandlingsresultat

5) att studera behandlingseffektivitet genom att sätta olika nyttovariabler (reducerad mortalitet och morbiditet, livskvalitet) i relation till samhällets och individens totala kostnader.

## 1.3 Andra frågeställningar

1) att studera om kirurgisk behandling ur mortalitetssynpunkt blir lönsammare ju svårare fetman är

2) att karaktärisera ändringar i kardiovaskulära riskfaktorer (blodtryck, cholesterol, triglycerider, Apo $A_1$, Apo B, Apo E, blodsocker, insulin, rökning) över 10 år hos medicinskt och kirurgiskt behandlade överviktiga patienter

3) att studera biverkningar vid medicinsk och kirurgisk behandling av fetma

4) att beskriva ändringar i läkemedelskonsumtion vid medicinsk och kirurgisk behandling av fetma

5) att med hjälp av registerstudien konkludera med vilken validitet resultaten från de två studerade grupperna kan generaliseras till att gälla alla överviktiga 37-57-åringar med BMI över givna gränser (män: BMI $\geq$36, kvinnor: BMI $\geq$40).

# 2 BAKGRUND

## 2.1 Associationer mellan fetmagrad och risk

Omfattande försäkringsstatistik (n = 1-5 milj) allt sedan seklets början har talat för ett samband mellan övervikt och mortalitet (1-4) medan populationsstudier av ordinär storlek (n = 1000-10000) och med måttliga observationstider som regel ej kunnat visa något samband mellan fetma och risk (för revy, se 5,6). Den statistiska associationen mellan kroppsbyggnad, morbiditet och mortalitet har under de senaste åren blivit definitivt fastställd genom tre nya arbeten: en ny försäkringsstudie (n = 5 milj) (7), en prospektiv icke-randomiserad studie sammanställd av "The American Cancer Society" (n = 0,75 milj) (8) samt en obligatorisk massundersökning ("The Norwegian Experience" (9)) som prospektivt följt praktiskt taget alla innevånare över 14 års ålder (n = 1,7 milj) i 17 av Norges 19 län. Såväl över- som undervikt är associaerade med riskökning i dessa tre studier. Risk - "body mass index" - sambandet är således kurvilinjärt och detta gäller för alla åldrar utom de allra högsta. Den relativa risken är dubblerad i U-kurvornas båda ändar och kurvornas form antyder ytterligare snabbt accelererande risk utanför det statistiskt säkerställda body mass index-intervallet. I "The Norwegian Experience" har man beräknat att populationens övervikt är ansvarig för 4,3% av all död hos män och kvinnor i åldersintervallet 30-79 år (9). Som jämförelse kan nämnas att lungcancer hos män eller bröstcancer hos kvinnor står för ca 4% av all dödlighet i samma norska åldersgruppeer. De viktigaste mortalitetsorsakerna vid övervikt är cerebro-cardiovaskulär sjukdom, diabetes och gallvägssjukdom. Coloncancer hos män samt bröst- och uteruscancer hos kvinnor är också överrepresenterade dödsorsaker vid fetma.

## 2.2 Associationer mellan fettvävsfördelning och risk

Vad övervikt beträffar har alla populations- och försäkringsstudier hittills varit inriktade på variabler som reflekterar graden av totalfetma (body fat, summa skinfold, body mass index etc). Emellertid har vi nyligen visat att fettvävens fördelning är av minst lika stor betydelse som mängden fettväv vid prediktion av kardiovaskulär risk.

I en transsektionell studie omfattande 930 överviktiga män och kvinnor visade vi att en android fettfördelning (hög midja/höftomkretskvot), oberoende av kön och fetmagrad, är associerad med högre blodtryck, högre serumtriglycerider, högre insulin och sämre glykostolerans än en gynoid fettvävsfördelning (låg kvot) (10). Midja/höft-omkretskvoten fanns tillgänglig som ingångsdata både i 1913 års män och i den göteborgska kvinnostudien. Nya beräkningar föranledda av vår transektionella studie (10) har visat att graden av android fettfördelning är en mycket starkare prediktor för kardiovaskulär morbiditet och mortalitet än sedvanliga mått på totalfetma. Detta gäller både män (11) och kvinnor (12). Hypoteser betr. orsaker till sambandet mellan fettvävsfördelning och risk återfinns i ref. 10 och 13.

## 2.3 Kauslitet och riskreversibilitet

De statistiskt säkerställda associationerna mellan övervikt, fettvävsfördelning och risk säger naturligtvis ingenting om fetmans

kausala betydelse för mortaliteten i ovan nämnda sjukdomar. Visserligen har ett par nyligen publicerade populationsstudier i långtidsuppföljningar kunnat visa att fetma är en av traditionella kardiovaskulära faktorer oberoende riskfaktor (12,14) men flertalet populationsstudier har ej kunnat finna detta (se ref. 5,6). För att bevisa ett kausalt samband mellan fetma och risk och för att belysa graden av riskreversibilitet krävs det studier som primärt intervenerar mot övervikt. Sådana kontrollerade studier har emellertid hittills aldrig genomförts, sannolikt därför att ingen metod med måttliga biverkningar under lång tid kunnat hålla tillräckligt många individer viktreducerade. Ventrikelingreppen erbjuder i detta sammanhang en möjlighet och den spontana svenska operationsvolymen är så stor att den bör utnyttjas för en kartläggning av den långsiktiga nyttan med viktreducerande kirurgi.

SOS avser att systematiskt studera om långvarig viktreduktion leder till minskad mortalitet och morbiditet. Eftersom midje/höft-kvoten visat sig vara en kraftfullare riskvariabel än olika index på totalfetma (11-12) kommer SOS att fästa avseende vid fetmasyndromets heterogenitet trots de principiella risker all subgruppering innebär (15).

## 2.4 Tidigare behandlingsstudier

### 2.4.1 Icke-kirurgisk behandling

Vid konventionell kalorirestriktionsbehandling är drop-out-frekvensen mycket hög och efter två år har mer än 90% av patienterna recidiverat i vikt (16). Beteendemodifierande behandling har förbättrat graden av viktnedgång men har som regel inte förbättrat långtidsresultaten (17,18). De hittills bästa beteendemodifierade resultaten har Björvell och Rössner rapporterat men inte ens i deras studie kvarstod mer än 10 procents viktreduktion efter 4 år (44). Fysisk träning som enda terapi ger ingen viktnedgång vid svår övervikt (19,20,21) och endast några få kilos viktnedgång vid måttlig övervikt (22,23). Compliance i fysiska träningsprogram är lika dålig som i bantningsprogram. Efter 6 månader har mer än 50% av patienterna givit upp (24) och efter 2 år finns högst 15% av de ursprungligen deltagande patienterna kvar i träningsprogrammen (25). Aptitnedsättande medel ger bättre korttidsresultat än placebo men långtidsresultaten under eller efter farmakologisk behandling är lika dåliga som för annan icke-kirurgisk terapi (för revy, se 26).

### 2.4.2 Kirurgisk behandling

Jejuno-ileal bypass (JIB) ger viktnedgång på 30% eller mera (27,28, 29) och det är väl dokumenterat att viktnedgången blir permanent (29,30). Biverkningssidan är emellertid allvarlig vid JIB. Den postoperativa mortaliteten ligger under 1% (0-30 dagar) men den operativa morbiditeten och de sena komplikationerna (artrit, njursten, leverskador, elektrolytrubbningar) är betydande (29). Cholesterol sänks drastiskt (29) men detta gäller både LDL- och HDL-cholesterol (28).

Gastric bypass operationer (GIB) ger en lika god viktnedgång som JIB (29,31-33). Den viktnedgång som uppnås i 2-5-årsuppföljningar ligger på 35-40% av initialvikten (31-39).

Horisontella gastroplastiker (GP) av olika typ ger 16-29% viktnedgång (34-36,39). Inga publicerade uppföljningstider är längre än 2 år. "Banding" ger samma eller något bättre resultat än GP (39).

Vertical banded gastroplasty (VBG) har följts under upp till 3 år och ger i genomsnitt 31% viktreduktion (38-40).

Operationsmortaliteten ligger i genomsnitt på 0,4, 0,5 och 0,7% för VBG, GIB och GP i serier startade efter 1978 (34-36,38).

Reoperationsfrekvensen är mycket hög (13%/år) vid banding och GP (38). Flertalet reoperationer företas på grund av bristande viktnedgång som i sin tur beror på stomadilatation (41). Vid GIB och VBG är den årliga revisionsfrekvensen mycket lägre, 2,8 resp. 1,7% (38).

Stomastenoser är vanliga vid GP (10%) men ej vid GIB (4%) eller VBG (1%). Beträffande övriga komplikationer är GIB och GP likvärdiga medan VBG ligger lägst (33-38). Jämfört med JIB är de sena komplikationerna färre och framför allt av beskedligare art vid såväl GIB, GP som VBG.

Även om långtidsuppföljningar (>10 år) ännu saknas förefaller det osannolikt att de viktnedgångsresultat som har uppnåtts med GIB och VBG skulle försämras drastiskt efter 3-5 år.

### 2.4.3 Konklusion betr. tillgängliga behandlingsstudier

Hittills tillgängliga icke-kirurgiska behandlingsmetoder är i stort sett verkningslösa på lång sikt. Flera kirurgiska metoder ger god viktnedgång i 3-5 årsuppföljningar men långtidsuppföljningar (>10 år) finns än så länge endast vad gäller JIB. Alla kirurgiska metoder är associerade med komplikationer men GIB och VBG i mindre grad än JIB och GP. Den ideala operativa metodiken har ännu ej fastställts.

# 3 ARBETSPLAN

## 3.1 REGISTERSTUDIEN

I samband med en landsomfattande, ev. kostnadsfri medicinsk undersökning av överviktiga kommer ett register över de undersökta att upprättas. Registret blir källan till de flesta kontroll- och kirurgfallen i interventionsstudien. Dessutom utgör registret en värdefull bakgrundsinformation t.ex. när det gäller att bedöma interventionsstudiens externa validitet. Registret kommer att omfatta 4000-10000 personer.

### 3.1.1 Patienturval.

Den medicinska hälsoundersökningen vänder sig till samma patientkategorier som Interventionsstudien, dvs. 37-57-åriga män med BMI ≥36 och 37-57-åriga kvinnor med BMI ≥40. Eftersom BMI torde vara ett alltför komplext rekryteringsbegrepp måste en kroppslängdstratifierad tabell användes som anger minimivikter för varje längdområde:

| Kvinnor 40-59 år | | Män 40-59 år | |
|---|---|---|---|
| Om Du har kroppslängden | får Du vara med i undersökningen om Du väger minst | Om Du har kroppslängden | får Du vara med i undersökningen om Du väger minst |
| cm | kg | cm | kg |
| - 159 | 90 | - 169 | 92 |
| 160 - 169 | 102 | 170 - 179 | 104 |
| 170 - 179 | 115 | 180 - 189 | 117 |
| 180 - | 130 | 190 - | 130 |

Utgående från antalet 40-59-åriga män (960000) och kvinnor (939000) i Sverige (1983) samt fördelningen av den norska populationen i olika ålders- och BMI-klasser uppskattas populationen som SOS initialt vänder sig till till minst 15 000 kvinnor och 7 000 män. Populationen kan vara betydligt mycket större än 22 000 personer eftersom "The Norwegian Experience" underskattar frekvensen av svår övervikt (9 och Hans Waaler, personligt meddelande). Av de ca 22 000 - 30 000 personer som Registerstudien vänder sig till uppfyller ca hälften förstahandskriterierna (se 3.3.2) för Interventionsstudien.

### 3.1.2 Rekryteringsmetoder

Patienterna nås genom information i Radio och TV samt genom annonser i flertalet dagstidningar i Sverige. Av program och annonser skall på ett lättfattligt sätt framgå att fetmasyndromet ur risksynpunkt är heterogent och att vissa överviktiga löper ökade risker att insjukna eller dö i komplikationer till fetman. En landsomfattande, ev. kostnadsfri medicinsk undersökning av kraftigt överviktiga genomföres därför. Av informationen skall även framgå att undersökningen om så önskas kan åtföljas av fetmabehandling.

Patienterna får till SOS-sekretariatet anmäla sitt intresse för den medicinska undersökningen på annonstalonger eller på anmälningsblanketter som skall finnas tillgängliga i primärvården. Vid anmälningen uppges namn, adress, 10-siffrigt personnummer samt telefon till bostad och arbete. Sekretariatet registrerar patienterna tillsänder dem enkätformulär och planerar in distriktsläkarbesök.

### 3.1.3 Information, frågeformulär och mätvariabler

SOS-sekretariatet tillsänder patienten allmän information om SOS-studien (blankett RM1) samt länsspecifik information (RM2). Vidare utsändes en huvudenkät (RE1), en kostenkät (RE3) och två psykologenkäter (RE4 och RE5). RE4 utgör underlag för matchning vad beträffar psykologiska variabler medan RE5 är en resultatenkät. Följdfrågor kan också komma att skicas ut. Påminnelserutiner finns inlagda. Sammantagna besvarar enkäterna frågor om aktuellt datum, civilstånd, hereditet, fetmans duration och svårighetsgrad samt tidigare och aktuella sjukdomar med fokusering på kärlkramp, hjärtinfarkt, slaganfall, claudicatio intermittens, diabetes, högt blodtryck och cancer. Vidare får patienten besvara frågor om fysisk aktivitet, rök-, alkohol- och kostvanor, aktuell medicinering, sjukhusvård när och var samt frågor om livskvalitet och kroppsuppfattning.

När all enkätinformation inkommit från patienten beställer SOS-sekretariatet tid åt patinten vid närmaste distiktsläkarmottagning samt tillsänder distriktsläkaren en enkät (RE21). Vid undersökningen fyller distriktsläkaren i RE21, som innehåller följande variabler: Läkarens namn, läkarmottagningens namn, adress och telefon, från id-kort kontrollerat 10-siffrigt personnummer på patienten, längd utan skor, vikt i underkläder, midje- och stussomfång enl. vissa preciseringar, blodtryck i liggande efter 5' vila samt fasteblodsocker (om reflektometer finns på mottagningen), Hb, SR, albustix och clinistix. Ett EKG tages. Nio stycken 10 ml rör fylles med blod för framställning av serum, plasma och heparinblod. Läkarens enkät, id-märkt EKG samt idmärkta rör med serum, plasma och blod sändes samma dag till SOS:s sekretariat. Delar av serum analyseras ankomstdagen vid Sahlgrenska sjukhusets Centrallaboratorium med avseende på elstatus, leverstatus, kreatinin, urat, chol, TG, HDL-cholesterol, insulin och glykos.

Mätvärden överföres automatiskt från C-labs dator till den av SOS utnyttjade datorn. Resterande blodprodukter fryses i 2 ml rör vid -80$^{o}$C. EKG:n värderas kliniskt samt arkiveras för interventionsstudien. Patient- och läkarenkäterna inmatas på data. Patienten och distriktsläkaren informeras skriftligen om undersökningsresultaten via automatisk brevutskrift.

Registreringsdatum i registerstudien blir läkarundersökningsdagen. Från detta datum inhämtas via enkät information om vikt efter 1/2, 1, 2 och 3 år. Total mortalitet kartlägges efter 10 år (fig. 1).

## 3.2 INTERVENTIONSSTUDIEN

3.2.1 Rekrytering. Patienterna i interventionsstudien rekryteras ur Registerstudien. Kirurgfall kan också rekryteras från befintliga väntelistor för gastroplastik vid kirurgklinikerna.

I Registerstudiens huvudenkät (RE1) har patienten fått svara på frågan om han/hon vill ha ytterligare information om kirurgisk behandling av fetma. I ett första steg tillsändes patienten i så fall ytterligare skriftlig information. Om patienten efter den skriftliga informationen fortfarande är intresserad av kirurgisk behandling av fetma bokar SOS-sekretariatet en tid åt patienten vid deltagande kirurgpoliklinik i länet.

I RE1 har också frågor ställts som visar om patienten är intresserad av att deltaga som kontrollfall i Interventionsstudien. Interventionsstudiens innebörd har förklarats för patienten i RM1.

3.2.2 Patienturval. För att begränsa studiens storlek kommer endast patienter med hög risk att inkluderas i studien. Åldersmässigt har 37-57-åringar valts. Yngre patienter med nedan föreslagna BMI-kriterier har en så låg absolut 10-årsrisk att gruppernas medelrisk skulle sänkas alltför mycket om de inkluderades. Studien bleve därmed orimligt stor. Äldre patienter anses av många kirurger ej lämpade för gastroplastik. Endast patienter med kraftig övervikt och därmed hög risk inkluderas.

Inklusionskriterierna baseras på body mass index (BMI, (vikt, kg)/(längd, m)$^2$ enligt:

1:a-handskriterier:

För män (37-57 år): BMI $\geq$36 kg/m$^2$

För kvinnor (37-57 år): BMI $\geq$40 kg/m$^2$

2:a-handskriterier (se nedan):

För män (37-57 år): BMI >34 kg/m$^2$

För kvinnor (37-57 år): BMI >38 kg/m$^2$

Ca 11000-15000 personer i Sverige beräknas uppfylla 1:a-handskriterierna för inklusion. Om färre än ca 2000+2000 fall kan inkluderas i interventionsstudien enl. 1:a-handskriterierna aktiveras 2:a-handskriterierna. Befolkningsunderlaget dubbleras därvid utan att medelrisken för inkluderade fall påverkas i någon större utsträckning.

Exklusionskriterier

1. Genomgången magsårsoperation (raphi undantages)
2. Verifierat magsår under de senaste 6 månaderna
3. Tidigare viktreducerande kirurgi (tandcerklage undantages)
4. Aktiv cancer under de senaste 5 åren
5. Hjärtinfarkt under de senaste 6 månaderna
6. Anamnes på frekvent (varje vecka) bulemiskt ätande

7. Alkoholproblem enl. patientens egen värdering. Alkoholkonsumtion motsvarande högre intag än 40 cl starksprit per vecka enligt kostenkät.
8. Psykiska besvär som begränsat arbetsförmågan under de sista 12 månaderna enl. patientsvar. Psykiska och/eller kooperativa besvär som enl. distriktsläkarens bedömning skulle göra "patienten definitivt oförmögen att klara kirurgisk behandling".
9. Handikapp som enl. distriktsläkarens bedömning "skulle göra det svårt för patienten att genomgå kirurgisk behandling".

Punkt 8 och 9 gäller såväl kirurg- som kontrollfall.

### 3.2.3 Dimensionering och observationstid

Den kirurgiskt behandlade gruppen antages i genomsnitt få en 30-procentig viktnedgång medan kontrollgruppens viktnedgång över 10 år blir obetydlig.

10-årsmortaliteten i kontroll- och kirurggrupperna antages bli 12 resp. 9%. För att med 5% signifikans och 80% styrka kunna påvisa skillnader mellan grupperna krävs det totalt 3300 individer i interventionsstudien. Som gardering mot statistiskt ogynnsammare utfall utformas studien enligt följande principer:

a) Den kirurgiska gruppens storlek höjs från den teoretiskt nödvändiga storleken på 1650 individer till approximativt 2000 individer (se dock c) och d) nedan).

b) Om antalet individer i registerstudien så tillåter matchas 2 kontrollindivider per varje kirurgindivid. Dubbelmatchningen skapar i sig ökade möjligheter att uppnå signifikans och utgör dessutom en skyddsmekanism mot oundviklig förlust av kontrollindivider genom att dessa kräver operation under studiens gång.

c) Om den tidiga operationsmortaliteten betr. de 1500 1:a kirurgfallen blir högre än 1% omdimensioneras studien under antagandena att kontrollfallen får 12% 10-årsmortalitet och kirurgfallen får (8% + tidig operationsmortalitet, %) 10-årsmortalitet. Inklusion av det teoretiska antalet nödvändiga fall plus ytterligare 700 fall avslutas om möjligt under det tredje inklusionsåret.

d) Studien har dimensionerats för 10 års observationstid men kortare eller längre observationstid bör tillämpas under vissa förutsättningar för att ge studien adekvat statistisk styrka:

- Om mindre än 2000 individer i kontrollgruppen: Studien pågår tills 240 dödsfall inträffat i kontrollgruppen.

- Om fler än 2000 individer i kontrollgruppen: Studien pågår tills 12% mortalitet har uppnåtts i kontrollgruppen.

### 3.2.4 Matchningsprocedur, inklusionsdatum och undersökningstillfällen

Alla i registret som förklarat sig intresserade av kirurgi remitteras till närmast deltagande kirurgiska klinik för bedömning.

Kirurgklinikerna anmäler till SOS-sekretariatet alla registerfall som bedöms lämpade för operation. Alla andra fall som redan finns eller som utan registerstudiens medverkan blir upptagna på väntelista för gastroplastik och som uppfyller inklusionskriterierna anmäles också till sekretariatet.

För varje opererat kirurgfall tillföres ett eller - om registerstudiens storlek så tillåter - två kontrollfall. Inklusionsdag för kirurg- och kontrollfallen blir kirurgfallets operationsdag.

Kontrollfallen väljes med en gruppmatchningsprocedur så att kontroll och kirurggruppernas medelvärden blir så lika som möjligt med avseende på kön, ålder, längd, vikt, midja/stuss-kvot, blodtryck, cholesterol, triglycerider, blodsocker, rökvanor samt psykosociala karaktäristika.

För fall uttagna till interventionsstudien bryts registerstudiens tidsplanering. Inklusionsdatum för kontroll- och kirurgfall blir kirurgfallets operationsdatum. Från detta datum räknat genomföres undersökningar av kontroll- och kirurgfallen efter 1/2, 1, 2, 3, 4, 6, 8 och 10 år. Vid 0 (preop.), 2 och 10 år insamlas förutom anthropometriska data också blod och urin för kemiska analyser.

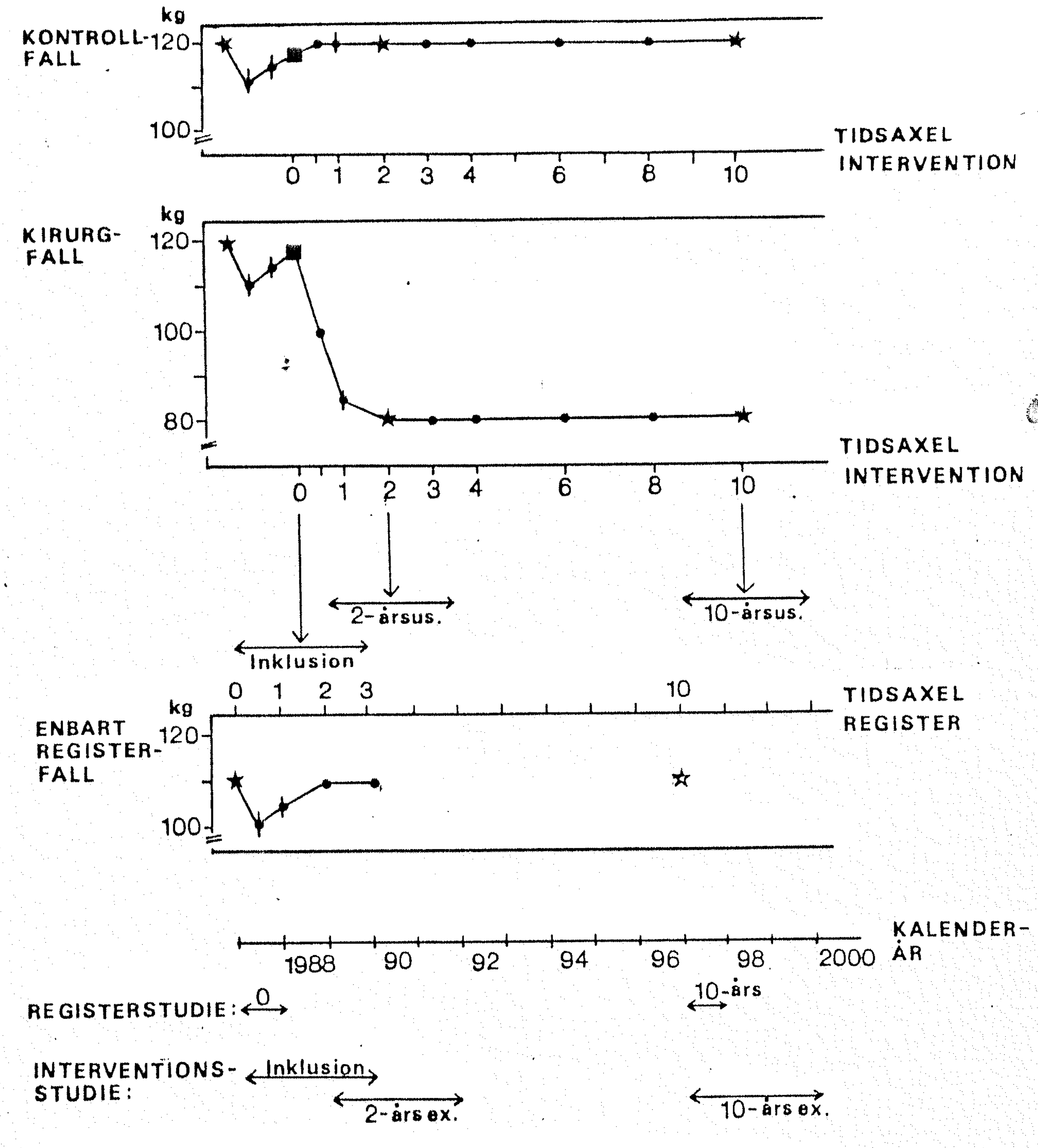


Fig. 1. Tidsmässig relation mellan kalenderår, register-, kirurg- och kontrollfall som förväntade förändringar i kroppsvikt.

- Initial undersökning samt för interventionsstudiens del 2- och 10-årsundersökningar.
- Kartläggning av mortalitet efter 10 år för registerfall som ej inkluderats i interventionsstudien
- Inklusion i interventionsstudien
- Viktregistrering
- Viktregistrering + psykologenkät
- Tidsintervall inom vilket indikerad händelse kan inträffa

3.2.5 Behandling

Kontrollfallen erbjudes kost- och motionsråd hos distriktsläkaren. Hypertoni, diabetes och hyperlipemi behandlas också enligt gällande standardrutiner vid resp. mottagning. SOS-sekretariatet avser ej att standardisera dessa rutiner.

Den kirurgiska behandlingen består av 2 typer av gastroplastiker, nämligen banding och vertical banded gastroplasty (VBG). För kliniker som väljer att bara utföra ett av ingreppen förordar sekretariatet VBG som är effektivast och samtidigt minst biverkningsbelastad. Om båda ingreppen utförs vid kliniken sker en randomisering genom SOS-datorns försorg.

## 3.3 REGISTRERING OCH VÄRDERING AV EFFEKTVARIABLER OCH BIVERKNINGAR

Längd. Uppmätes till närmaste cm på alla i samband med registrering.

Vikt. I Registerstudien registreras vikten initialt samt efter 1/2, 1, 2 och 3 år. Information om vikter måste insamlas betr. alla registrerade under hela Interventionsstudiens inklusionsperod eftersom man inte på förhand vet vilka som blir inkluderade. I Interventionsstudien registreras vikten vid inklusion samt efter 1/4, 1/2, 1, 2, 3, 4, 6, 8 och 10 år efter inklusionen.

Omfångsmått. Bålomfånget mätes i liggande på 3 olika nivåer: a) mitt emellan proc. enciformis och nedre laterala delen av thorax. b) mitt emellan nedre laterala delen av thorax och crista iliaca. c) i symfys-trochanter höjd.

Blod och urinprov. Alla Registerstudiens fall undersökes initialt med avseende på Hb, SR, albustix och clinistix, triglycerider, cholesterol, HDL, serumglykos, seruminsulin, elstatus, leverstatus, kreatinin, urat och TSH.

Några veckor före planerad operation (Inklusionsundersökning) samt vid två och 10 år omundersökes alla fall i Interventionsstudien med avseende på alla ovan nämnda analyser plus apo $A_1$, apo B och apo E samt 24 timmars urinutsöndring och urea, kväve, Na, Cl, K och katekolaminer. Provtagningen ombesörjes av primärvården betr. kontrollfall och av kirurgklinik betr. kirurgfall.

Hb, SR, albustix och clinistix utföres vid primärvårds- eller kirurgmottagningen (redovisas i av läkare ifylld enkät) medan alla andra analyser utföres vid C-lab. (Lindstedt) eller Utvecklingslab (Bondjers), SS. Lab.resultat överföres automatiskt från C-labs till SOS-sekretariatets dator.

Blodtryck och EKG. Blodtryck mätes i liggande ställning efter EKG-tagning och ytterligare 5 minuters vila. Diastoliskt blodtryck registreras i fas V. Sedvanligt 12 avledningars EKG tages. Blodtryck registreras vid alla undersökningar medan EKG tages vid Registerundersökning, Inklusionsundersökning, 2- och 10-årsundersökningarna. EKG analyseras initialt för kliniskt rutinbruk och senare enl. modifierad Minnesota-kod.

Psykologiska testformulär. Enkät RE4 infylles av alla registerfall och användes för senare ev. matchning av kirurg- och kontrollgrupper. Enkät E5 är en resultatenkät som användes vid alla undersökningstillfällen i studien.

Rök-, alkohol-, kost- och motionsvanor samt läkemedelskonsumtion. Information insamlas vid alla undersökningstillfällen.

Sjukskrivning och pensionering uppdateras betr. Interventionsstudiens fall från FK:s centraldator.

Morbiditet. Förekomster och insjuknande i hypertoni, angina pectoris, claudicatio intermittens, stroke, hjärtinfarkt, diabetes och gallvägssjukdom registreras kontinuerligt under uppföljningen. Ändpunkterna är definierad i appendix.

Död. Information om död sammanställes av styrkommittén (se punkt 7) först efter 10 år men insamlas kontinuerligt. Ingående kirurgkliniker och behandlande distriktsläkare skall kontinuerligt rapportera om inträffade dödsfall inom interventionsstudien. De patienter som ej nås efterforskas via States person- och adressregister. Varje år samkörs avlidnas personnummer i interventionsstudien mot Statistiska Centralbyråns dödsorsaksregister. Journaler rekvireras vid behov.

## 3.4 STATISTIK

Undersökningsstorleken är baserad på att ett tvåsidigt test och signifikansnivån 5% kommer att användas. Sannolikheten att med testet upptäcka en skillnad i mortalitet mellan kontroll- och kirurggrupp har satts till 80%. Detta innebär att styrkan i studien är 80% och felet av andra slaget är 20%. Olika antagande om ändringen i 10-årsmortaliteten i de båda grupperna har gjorts utgående från Waaler (9). Den uppskattade 10-årsmortaliteten i en longitudinell studie med 60% kvinnor (BMI $\geq$40) och 40% män (BMI $\geq$36) har beräknats till 13%. Reduktion av övermortalitet definieras som $(r_1-r) \times 100/(r_1-r_0)$ där $r_1$ = 13%, $r_0$ = 6,4% och r = estimerad 10-årsmortalitet.

### Regler för interimsanalyser

Säkerhetskommittén (se punkt 11) följer oberoende av styrkommittén mortalitetsutvecklingen. Studien avbrytes endast vid exceptionellt olika utveckling av mortalitet inom de två grupperna. En högre mortalitet i den kirurgiska gruppen förutses under de två första åren.

### Analys

1. Jämförelse av ingångskaraktäristika mellan den opererade gruppen och kontrollgruppen. Om det föreligger betydande skillnader mellan grupperna beträffande några ingångskaraktäristika och om dessa variabler också är signifikant relaterade till den studerade ändpunkten kommer dessa variabler att behandlas som "confounders" i analysen (se 2).

2. Mantel's test kommer att utnyttjas för analys av skillnaden i mortalitetsutvecklingen mellan behandlingsgrupp och kontrollgrupp. Eventuellt göres stratifikation enligt punkt 1.

3. Konventionella test användes i övrigt vid andra jämförelser mellan grupperna.

## 3.5 SPECIELLA ASPEKTER

SOS erbjuder unika tillfällen att tvärvetenskapligt analysera olika aspekter av fetmaproblematiken. Till SOS har därför knutits ekonomisk, psykologisk och genetisk expertis.

3.5.1 Ekonomiska aspekter. En s.k. cost-effectivenessanalys skall genomföras inom ramen för detta projekt. Prof. Egon Jonsson vid Spri och Karolinska Institutet blir huvudansvarig för denna del av studien.

Alla direkta och indirekta kostnader för kirurgiska och medicinska åtgärder skall analyseras på ett representativt urval av patienterna. Urvalet blir beroende av den grad av detaljredovisning som finns inom olika landsting avseende kostnadsfördelning. Avsikten är att studien skall kunna redovisa den genomsnittliga kostnaden, inkl. olika spridningsmått, per patient och per typ av åtgärd. Effectiveness-sidan mäts både i monetära och i kvalitativa termer. Förutom att somliga medicinska variabler belyses i ekonomiska termer skall sjukskrivningsfrekvenser och sjukpensioner registreras. Patienternas och de anhörigas eventuella privatekonomiska uppoffringar samt ekonomiska konsekvenser som är relevanta för sjukvårdshuvudmannen beskrives. Hit hör exempelvis förändringar i utnyttjandet av sluten och öppen vård, räknat inte enbart i vårddagar och besök utan även i form av tester, provtagningar, laboratorieundersökningar och andra belastningar på medicinsk service omräknat i ekonomiska termer. Slutligen skall cost-effectivenessanalysen också beskriva de samhällsekonomiska konsekvenserna av handläggning enl. kirurg- och kontrollförfarandet.

3.5.2 Psykologiska aspekter. Dessa studier utförs i samarbete med prof. Lennart Sjöberg och docent Marianne Sullivan vid Psykologiska Institutionen, Göteborgs Universitet.

Kirurg- och kontrollgruppen karaktäriseras initialt med avseende på ett antal fysiologiska (kön, ålder, längd, vikt, glykos, insulin etc) och psykologiska variabler.

Ingångsvariabler av prediktivt värde för viktnedgång och recidivbenägenhet fastställes genom analys av hela interventionsmaterialet. Psykologiska mekanismer i samband med viktrecidiv beskrives i representativa stickprov.

Sambanden mellan ingångvariabler och förändringar i kroppsvikt, kroppsuppfatting och livskvalitet analyseras med hjälp av statistiska metoder för kausalanalys (s.k. LISREL-modeller). Härvid uppmärksammas säskilt om viktreduktion medierar förändringar i livskvalitet eller om de båda variablerna enbart samvarierar på grund av den gemensamma behandlingskomponenten.

3.5.3 Genetiska aspekter. De genetiska analyserna utföres i samarbete med prof. Claude Bouchard, Laval-universitetet, Québec, Canada.

Mycket kortfattat avser den genetiska analysen att belysa i vad mån arv och i vad mån miljöpåverkan bestämmer grad av fetma (analys av

hela registerstudien) och i vad mån hereditär och icke-hereditär influens påverkar behandlingsresultatet (analys av interventionsstudien). Med andra ord möjliggör SOS analyser som belyser genotypens betydelse för aktuell vikt och för viktförändringar.

Grad av viktöverensstämmelse bland biologiska och icke-biologiska släktingar över tre generationer (föräldrar, syskon och sammanboende, barn) analyseras med CORRFAM och ANOFAM samt med "path-analysis procedures" (TAU, XTAU and BETA). Dessa metoder ger mått på familjär anhopning av fetma, sammanlevnadseffekter, transmissionseffekter mellan generationer, maternella och paternella effekter samt uppskattningar av hereditära och miljömässiga inflytanden. Genom att sätta behandlingsresultatet i relation till vuxenvikterna hos biologiska och icke-biologiska släktingar kan man med liknande tekniker analysera i vad mån individens genotyp bestämmer terapiutfallet.

De obesitasdata som finns tillgängliga betr familjär anhopning, maternala och paternala effekter, totala transmissionseffekter och biologiskt arv är utomordentligt bristfälliga. Undersökningar som belyser genotypens betydelse för viktförändringar saknas helt. SOS erbjuder en unik möjlighet att studera dessa frågeställningar i ett mycket stort material av kraftigt överviktiga individer. Resultaten kan bli av stor betydelse både för förbättrad prevention och behandling av fetma och för framtida forskning om fetman och dess metaboliska komplikationer.

## 4 ETISKA ÖVERVÄGANDEN

Sammanfattning av de etiska aspekterna på SOS:

Tidigarelagd död pga operationsmortaliteten balanseras av minskad övrig mortalitet inom 1,3 år efter inklusion i Interventionsstudien.

Kirurgisk behandling räddar 60-80 liv över 10 år under förutsättning att dimensioneringsantagandena visar sig riktiga

Det är på förhand omöjligt att veta om dimensioneringsantagandena är riktiga. Det är således etiskt välmotiverat att jämföra det kirurgiska behandlingsresultatet med mortalitetsutfallet i den rutinmässigt omhändertagna kontrollgruppen trots att mortaliteten i den senare gruppen beräknas ligga på 12% per 10 år.

SOS systematiserar kirurgisk fetmabehandling som under alla omständigheter pågår spontant i Sverige. Det vore därför, som projektledningsgruppen ser det, närmast oetiskt att avstå från möjligheten att utreda om kirurgisk behandling av fetma leder till reducerad morbiditet och mortalitet.

SOS ger möjlighet att strikt randomisera mellan två kirurgiska tekniker vid flera deltagande centra. En mera omfattande och därmed pålitligare värdering av optimal operationsteknik kommer därför till stånd om SOS genomföres.

Sverige har ur global synpunkt ett speciellt ansvar eftersom vårt land är ett av ett fåtal länder i vilket en studie av denna typ över huvud taget kan genomföras.

## 5 SAMHÄLLSEKONOMISKA KONSEKVENSER

Sjukvårdskostnaderna i sig för projektet uppgår till 70 a 80 milj kronor. Av dessa kostnader är ca 20 milj. extra kostnader på grund av studien medan övriga kostnader är sådana som samhället under alla omständigheter har för såvitt inte viktreducerande kirurgi förbjudes i riket. Över en 13-årsperiod blir de extra kostnaderna ca 1 miljon kronor för ett medelstort landsting. Detta är kostnader som pilotlänen funnit rimliga i förhållande till de potentiella vinster som projektet innebär. Under inklusionsperioden på 3 år krävs i vissa landsting omprioriteringar varvid patientgrupper utan nämnvärda riskförhöjningar (varicer, bråck, okompl. gallor) drabbas av förlängda väntetider till operation.

## 6 STUDIENS ADMINISTRATION

SOS får följande organisatoriska struktur

. Styrgrupp

Doc. Lars Sjöström (ordf., försöksledare)
Prof. Calle Bengtsson, Allmänmed. Inst, Göteborg
Doc. Lars Backman, Kir.klin., Danderyd
Prof. Claude Bouchard, Laval University, Québec, Canada
Prof. Egon Jonsson, Ekonom, Stockholm
Dr. Ingemar Näslund, Kir.klin., Örebro
Doc. Lars Olbe, Kir.klin. II, SS, Göteborg
Prof. Lennart Sjöberg, Psykol. Inst., Gbg:s Universitet
Doc. Marianne Sullivan, Psykol. Inst., Gbg:s Universitet

. Säkerhetskommitté

Prof. Sven Dahlgren, Kir.klin., Umeå
Doc. Bo Larsson, Med.klin. I, SS, Göteborg
Prof. Hans Wedel, Bio-statistiker, Nordiska hälsovårdshögskolan, Göteborg

. Referensgrupp

Styrgruppens och säkerhetskommitténs medlemmar plus 1 representant från varje ingående kirurgkilnik och 1 representant från varje landstings primärvård.

. Kemisk analysenhet. Prof. Sven Lindstedt, Institutionen för Klinisk Kemi, Göteborg har åtagit sig att mot kostnadstäckning och i samarbete med doc Göran Bondjers, Med. I, SS utföra alla kemiska analyser.

## 7 PILOTSTUDIE

Med start våren 1987 genomföres en pilotstudie i Örebro län, Kronobergs län samt Göteborgs stad). Brister i rutiner, formulär och information uppdagade under pilotstudien korrigeras så långt detta är möjligt.

Pilotstudiens målsättning är

att erhålla information om det antal Registerfall per 100 000 invånare som kan nås genom en kampanj i lokala massmedia

att beskriva primärvårdens belastning på grund av Registerstudien

att erhålla information om hur stor andel av Registerfallen som är intresserade av och lämpade för kirurgisk behandling av fetma

att beskriva korttids (3 månader) morbiditet och mortalitet associerade med viktreducerande ventrikelkirurgi i Sverige

att med hjälp av ovanstående kunskaper ta fram underlag för beslut om en eventuell landsomfattande SOS-studie, samt

att trimma in SOS-studiens tämligen komplicerade data-administrativa rutiner inför en eventuell landsomfattande studie.

## 8 REFERENSER


1. Medico-Actuarial Mortality Investigation. New York, N.Y., Association of Life Insurance Medical Directors and Actuarial Society of America, 1913.

2. Ideal Weight for Women. Statistical Bulletin. New York, N.Y., Metropolitan Life Insurance Co, October 1942.

3. Ideal Weight for Men. Statistical Bulletin. New York, N.Y., Metropolitan Life Insurance Co., June 1943.

4. Build and Blood Pressure Study, 1959, volume 1, Chicago, IL, Society of Actuaries, 1959.

5. Keys, A., C. Aravanis, H. Blackburn, R. Buzina et al (1980): Seven countries: a multivariate analysis of death and coronary heart disease. Cambridge, Mass. Harvard University Press.

6. Andres, R. Effect of obesity on total mortality. Int. J. Obesity 4:381-386, 1980

7. Build Study 1979. Society of Actuaries and Association of Life Insurance Medical Directors of America.

8. Lew, E. and L. Garfinkel. Variations in Mortality by weight among 750.000 men and women. J. Chron. Dis. 32:563-576, 1977.

9. Waaler, H. Height, Weight and Mortality. The Norwegian Experience. Acta Med. Scand. Suppl. 679, 1983.

10. Krotkiewski, M., Björntorp, P., Sjöström, L. & Smith, U. Impact of obesity on metabolism in men and women - importance of regional adipose tissue distribution. J. Clin. Invest. 72:1150-1162, 1983.

11. Larsson, B., Svärdsudd, K., Welin, L., Wilhelmsen, L., Björntorp, P, Tibblin, G. Abdominal adipose tissue distribution, obesity and risk of cardiovascular disease and death. A 13-year follow-up of the study of men born in 1913. Brit. Med. J. 288:1401-4, 1984.

12. Lapidus, L., Bengtsson, C., Larsson, B., Pennert, K., Rybo, E. & Sjöström, L. Distribution of adipose tissue and risk of cardiovascular disease and death: a 12-year follow-up of participants in the population study of women in Gothenburg, Sweden. Brit. Med. J. 289: 1257-1261, 1984.

13. Björntorp, P. & Sjöström, L. Adipose tissue dysfunction and its consequences. In: New Perspectives in Adipose Tissue. Eds. A. Cryer and R. Van. Butterworths, in print 1985.

14. Hubert, H., M. Feinleib, P. McNamara & W. Castelli. Obesity as an Independent Risk Factor for Cardiovascular Disease: A 26-year Follow-up of Participants in the Framingham Heart Study. Circulation 67: 968-977, 1983.

15. Pocock, S. Current issues in the design and interpretation of clinical trials. Brit. Med. J. 290:39-42, 1985.

16. Krotkiewski, M., Sjöström, L., Björntorp, P., Carlgren, G., Garellick, G. & Smith, U. Adipose tissue cellularity in relation to prognosis for weight reduction. Int. J. Obesity 1:395-416, 1977.

17. Stunkard, A.J. & Penick, S.B. Behavior modification in the treatment of obesity. Arch. Gen. Psych. 36:801-806, 1979.

18. Brightwell, D.R. & Sloan, C.L. Long-term results of behavior therapy for obesity. Behav. Ther. 8:898-905, 1977.

19. Björntorp, P., de Jounge, K., Sjöström, L. & Sullivan, L. Physical training in human obesity. II. Effects on plasma insulin in glucose intolerant subjects without marked hyperinsulinemia. Scand. J. Clin. Lab. Invest. 32:41, 1973.

20. Björntorp, P., de Jounge, K., Krotkiewski, M., Sullivan, L., Sjöström, L. & Stenberg, J. Physical training in human obesity. III. Effects of long-term physical training on body composition. Metabolism 22:1467, 1973.

21. Krotkiewski, M., Sjöström, L. & Björntorp, P. Physical training in hyperplastic obesity. V. Effects of atropine on plasma insulin. Int. J. Obesity 4:49-56, 1980.

22. Björntorp, P., Grimby, G., Sanne, H., Sjöström, L., Tibblin, G. & Wilhelmsen, L. Adipose tissue fat cell size in relation to metabolism in weight stabile, physically active men. Horm. Metab. Res. 4:182, 1972.

23. Krotkiewski, M., Mandroukas, K., Sjöström, L., Sullivan, L., Wetterqvist,H. & Björntorp, P. Effects of long-term physical training on body fat, metabolism, and blood pressure in obesity. Metabolism 28:650-658, 1979.

24. Dishman, R.K. Compliance/adherence in health-related exercise. Health Psychology 1:237-267, 1982.

25. Katahn, M. & McMinn, M.R. Obesity: A biobehavioral point of view. Paper presented at the New York Academy of Science. November 1982.

26. Blundell, J.E. & Rogers, P.J. Pharmacologic Approaches to the understanding of obesity. Psychiatric Clinics of North America 1:629-650, 1978.

27. Kral, J.G., Björntorp, P., Scherstén, T. & Sjöström, L. Body composition and adipose tissue cellularity before and after jejuno-ileostomy in severely obese subjects. Europ. J. Clin. Invest. 7:413-419, 1977.

28. Kral, J., Sjöström, L. & Gustafson, L. Effects of jejuno-ileal bypass on serum lipoproteins and glucose tolerance in severely obese patients. Europ. J. Clin. Invest. 10:363-367, 1980.

29. Rucker, R., Chan, E., Horstmann, J. et al. Searching for best weight reduction operation. Surgery 96:624-631, 1984.

30. Wiklund, B. Fate of weight: Ten-year observation after jejuno-ileal bypass for obesity. Acta Chir. Scand. 148:443-52, 1982.

31. Buckwalter, J.A. Clinical trial of jejuno-ileal and gastric bypass for the treatment of morbid obesity. Am. Surg. 46:377-81, 1980.

32. Griffen, W.O. Table 4-1. In: Mason, E.E. ed: Surgical treatment of obesity. Philadelphia 1981, W.B. Saunders Co, p. 143.

33. Joffe, S.N. A review. Surgery for morbid obesity. J. Surg. Res. 33: 74-88, 1982.

34. Lechner, G:W. & Callender, A.K. Subtotal gastric excluson and gastric partitioning: A randomized prospective comparison of one hundred patients. Surgery 90:637-42, 1981.

35. Laws, H.L., Piantadosi, S. Superior gastric reduction procedure for morbid obesity. Ann. Surg. 193:334-6, 1981.

36. Piores, W.J., Flickinger, E.G., Melheim, D. et al. The effectiveness of gastric bypass over gastric partition in morbid obesity. Ann. Surg. 196:389-97, 1982.

37. Thompson, W.R., Amaral, J.F., Caldwell, M.D., Martin, H.F. & Randall, H.T. Complications and weight loss in 150 consecutive gastric exclusion patients. Am. J. Surg. 146:602-612, 1983.

38. Mason, E.E., Lewis, J.W., Doherty, C., Rodriquez, E.M., Scott, D.H. & Blommers, T. Vertical banded gastroplasty for morbidy obesity at three years. Manuskript.

39. Näslund, I. Personligt meddelande.

40. Mason, E.E. Vertical banded gastroplasty for obesity. Arch. Surg. 117:701-6, 1982.

41. Olsson, S.Å., Nilsson-Ehle, P., Pettersson, B.G. & Sörbris, R. Gastroplasty as a treatment for Massive Obesity - A Clinical and biochemical evaluation. Scand. J. Gastroenterology, in press.

42. Pocock, S.J. Clinical Trials. John Wiley, New York 1983.

43. Halperin, M., Rogot, E., Guarian, J. & Ederer, F. Sample sizes for medical trials with special reference to long-term therapy. J. Chronic Dis. 21:13-24, 1968.

44. Björvell, H. Treatment of severe obesity. Long-term follow-up, personality triats, eating behaviour and effects of peroral glycerol in obese subjects. Diss., Karolinska Institute, Stockholm, 1985.

45. Bouchard, C., Savard, R., Després, J.P., Tremblay, A., Leblanc, C. Body composition in adopted and biological siblings. Hum. Biol. 57: 61-75, 1985.

46. Rice, J., Cloninger, C.R., Reich, T. Multifactorial inheritance with cultural transmission and assortative mating. 1. Description and basic properties of the unitary models. Am. J. Hum. Genet. 30:618-643, 1978.

47. Rice, J., Cloninger, C.R., Reich, T. General causal models for sex differences in the familial transmission of multifactorial traits: an application to human spatial visualizing ability. Social Biol. 27:36-47, 1980.

48. Cloninger, C.R., Rice, J., Reich, T. Multifactorial inheritance with cultural transmission and assortative mating. Am. J. Hum. Genet. 31: 176-198, 1979.

APPENDIX

ÄNDPUNKTSDEFINITIONER

. Död oberoende av orsak är studiens primära ändpunkt.

Vid analys av betydelsen av viktreduktion inräknas alla dödsfall fr.o.m. dag 61 t.o.m. studiens slut.

Vid analys av betydelsen av kirurgisk behandling beaktas all dödlighet fr.o.m. inklusionsdagen.

. Kardiovaskulär morbiditet:

Hypertoni: Hypertoni föreligger då patienten antingen har pågående blodtrycksbehandling eller blodtryck systoliskt >160 och/eller diastoliskt >95.

Angina pectoris: Angina pectoris föreligger när patienten svarar ja på frågan: Får Du smärtor, tryck eller obehag i bröstet när Du går uppför backar eller trappor eller när Du går fort på slät mark?, samt svarar inom 10 min på frågan: Om bröstsmärtorna försvinner när Du stannar, hur snart försvinner de?

Claudicatio intermittens: Claudicatio intermittens föreligger om patienten har nedan angivna svarskombination på tre frågor:

| | fråga | svar |
|---|---|---|
| 1. | Brukar Du få smärtor i vaderna när Du går i uppförsbackar, trappor eller på plan mark? | JA |
| 2. | Om Du stannar, försvinner smärtan i vaderna då? | JA |
| 3. | Om smärtorna i vaderna försvinner när Du stannar, hur snart försvinner smärtorna? | Inom 5 min |

Stroke: Stoke anses föreligger om patienten svarar ja på frågan Har Du sedan föregående enkät varit intagen på sjukhus i samband med blödning eller propp i hjärnan? samt om journaluppgifter talar för akut cerebrovaskulär sjukdom enligt någon av nedanstående definitioner:

Hemorrhagia cerebri (Intracerebral blödning): Blödning in i hjärnparenchymet. Oftast akut insjuknande med huvudvärk och illamående. I regel hemisymtom, ev. med afasi, samt sänkt medvetandegrad. Vanligen hypertoniker. Likvor blodig (500 röda/$mm^3$). Eko-enc visar ofta överskjutning redan från början. CT-skalle visar typisk bild.

Thrombosis cerebri (Hjärninfarkt): Utveckling eller närvaro av trombos i en intracerebral artär. Vanligen "halvakut" insjuknande, i regel hemisymtom, ev. afasi, samt ofta obetydlig medvetanderubbning. Likvor utan röda. Eko-enc utan överskjutning från början. Hjärnstamsinfarkt (Mb Wallenberg) tas också med här, även utan att LP har utförts. CT-skalle sent i förloppet visar typisk bild.

Ocklusio arterial praecerebralis: som thrombosis cerebri, men angiografi av extrakraniella kärl har utförts och visat ocklusion.

Embolia cerebri: Plötslig blockering av cerebral artär av koagel eller annat material ditfört av blodströmmen. Urakut insjuknande med fokala neurologiska bortfallssymtom, i regel hemisymtom, och ofta utan medvetanderubbning. Hjärtat ofta embolikälla, t.ex. infarkt eller förmaksflimmer. Likvor utan röda.

Morbus crerbrovascularis acutus male definitus (Apoplexia NUD): Akut vaskulär insult enl. ovanstående definition men utredning har ej utförts som ger underlag för någon av ovanstående diagnoser. Bl.a. ingen LP eller CT-skalle. Eko-enc och EEG ger ej underlag för typdiagnos. Detta vanligaste diagnosen hos äldre.

Hjärtinfarkt:

Dödande hjärtinfarkt: Död i hjärtinfarkt enligt definitionerna möjlig eller definitiv hjärtinfarkt enligt nedan eller plötslig död där man vid obduktionen finner tecken till akut hjärtinfarkt eller avancerad coronararterioscleros utan annan förklaring till döden.

Icke-dödande infarkt:

Möjlig infarkt: Bröstsmärta av minst 15 min duration + något av följande kriterier:

a) tillkomst av T-vågsinversion i åtminstone 2 av 12 standard-EKG-avledningar utan förhöjda enzymvärden över normalvärdet.

b) endast ett förhöjt enzymvärde över normalvärdet

c) tillkomst av ny Q-våg eller förlust av R-våg eller ST-segmenthöjning efterföljd av T-vågsinversion i endast en avledning av standard-EKG:t.

Definitiv infärkt:

2 av de 3 följande kriterierna skall vara fullföljda:

a) bröstsmärta av åtminstone 15 min duration

b) åtminstone 2 värden över normalvärdet för enzym använt för hjärtinfarktdefinition

c) tillkomst av ny Q-våg eller förlust av R-våg eller ST-segmenthöjning efterföljd av T-inversion i åtminstone 2 avledningar i standard-EKG.

Diabetes

Diabetes anses föreligga om patienten har pågående behandling med peroral antidiabetika eller insulin och/eller fasteblodsocker över 7,0 mmol/l

Gallvägssjukdom

Gallvägssjukdom anses föreligga om patienten svarar ja på frågan: Har du eller har du haft bukbesvär som berott på eller misstänkts bero på gallsten? samt ja på frågan: Visade ultraljudsundersökning eller röntgenundersökning av gallblåsan att denna innehöll gallsten? eller ja på frågan: Har du haft inflammation i gallblåsan, s.k. cholecystit? eller ja på frågan: Är din gallblåsa borttagen?

# Swedish Obese Subjects (SOS)

Translation of original study protocol (19870113)

## Aim

The primary aim of the study is to investigate whether the increased mortality associated with obesity is reduced by weight loss. Gastroplasty surgery is used to achieve a sustained weight loss. Secondary aims include to investigate whether weight reduction is associated with reduced mortality and morbidity related to cardiovascular disease, diabetes and gallbladder disease. The study will also explore the importance of inheritance and environment for degree of obesity and weight loss results. Psychological responses of importance for low therapy effect and changes in quality of life during treatment will be analyzed. Finally, measures of efficacy will be applied to surgery and control groups. Thereby, different beneficial changes (reduced mortality and morbidity, quality of life etc) will be analyzed in relation to the costs for individuals and society.

## Background

A u-shaped association between body weight and mortality has been shown in several studies. In recently published papers we have shown that type of adipose tissue distribution is a stronger predictor for mortality and morbidity than the total degree of obesity. Controlled intervention studies in obese patients have never been performed. The causality of obesity is therefore uncertain for cardiovascular morbidity, diabetes and death and the possible risk reductions associated with weight loss not proven. All non-surgical obesity treatments are associated with very poor long-term results whereas some types of ventricular surgical procedures result in good weight loss and an acceptable rate of side effects.

## Work plan

### The registry study:

In a medical investigation, performed at primary health care centers nationwide, information and measures from 7000-10000 morbidly overweight individuals are collected. The collected variables include height, weight, measures of circumference, blood glucose, insulin, triglycerides, cholesterol, HDL, electrolyte status, urate, liver status, EKG, blood pressure, hereditary obesity, psychosocial- and economic factors as well as current and past illnesses. The registry forms the main recruitment base for the intervention study and is also valuable as an information source when assessing the external validity of the intervention study.

### The intervention study:

Patients from the registry study who have declared an interest in surgical treatment are scheduled for appointments at surgical departments for further information and examinations. Around 20 Swedish public surgical departments participate in the study. For every surgery case, one or – if the size of the registry study allows- two control cases is/are added. The day of inclusion for the surgery- and the control is the day of surgery for the surgery patient. Controls are selected with a minimizing method so that the mean values for

the control- and surgery groups are as similar as possible with respect to 17 factors with important prognostic value.

Surgery cases are followed regularly at their respective surgical departments and controls within primary care. The study will include 2000 surgery cases and 2000-4000 control cases. After an inclusion period of approximately 4 years, surgery and control cases are followed for 10 years.

### Effect registration and analysis:

The study is managed by computerized administration. Mortality as well as sick leave and retirement are captured by crosschecking with registers at Statistiska Centralbyrån (Statistics Sweden) and Försäkringskassan (the Swedish Social Insurance Agency). Weight changes and morbidity in the groups are collected through questionnaires at ½, 1, 2, 4, 6, 8, and 10-year follow-ups. Diagnoses associated with deaths and with hospitalizations are collected from death certificates and hospital records. When performing comparisons of mortality between surgery and control groups, death rates are calculated from the day of inclusion. When assessing the importance of weight loss, death rates are calculated from day 61 after day of inclusion.

Degree of weight concordance among biological and non-biological relatives over three generations will be analyzed using "path-analysis procedures" in collaboration with the Laval Institute in Québec. These methods provide measures of familial hereditary obesity, cohabitation effects, transmission effects between generations, maternal and paternal effects and assessments of hereditary and environmentally important factors. Psychological variables are analyzed using for example statistical methods for causality analysis (LISREL methods).

## Importance

The study has great medical, social and socioeconomic importance since mortality attributed to overweight is of the same magnitude (4%) as lung cancer in men and breast cancer in women.

## Research Program Swedish Obese Subjects (SOS)

A registry study and a prospective controlled intervention study of overweight using gastroplasty surgery as means for achieving weight loss and less likelihood of recidivism.

1. AIM
   SOS is partly a registry study and partly a prospective, matched intervention study in which a control group is treated by usual obesity care in primary care and a group surgically treated by some kind of ventricular surgical procedure at one of the 20 participating surgical departments.
   The aims are:
   1.1. <u>Primary aims</u>
      1. To investigate whether <u>weight reduction</u> is associated with a decrease in total mortality.
      2. To investigate whether <u>surgical treatment</u> is associated with a reduction in total mortality.

1.2. Secondary aims

1. To investigate whether weight reduction
   a. Is associated with reduced mortality and morbidity in cardiovascular disease (hypertension, angina, intermittent claudication, stroke and myocardial infarction), diabetes and gall bladder disease.
   b. Is associated with different rates of risk reduction in patients with android and gynoid adipose tissue distribution
2. To investigate whether surgical treatment
   a. Can reduce mortality and morbidity in cardiovascular disease (hypertension, angina, intermittent claudication, stroke and myocardial infarction), diabetes and gall bladder disease.
   b. Is associated with different rates of risk reduction in patients with android and gynoid adipose tissue distribution.
3. Psychological responses are investigated through
   a. Studies of variations in quality of life and body perception in control and surgery groups
   b. Finding psychological variables of predictive value for treatment results
   c. Studies on the psychology behind weight recidivism
4. To study the importance of genetic inheritance, environment during childhood and adulthood for degree of obesity and treatment results
5. To study treatment efficacy by relating benefit variables (reduced mortality and morbidity, quality of life) with the total costs for the individual and society

1.3. Other issues of investigation

1. To study if surgical treatment is more cost efficient for severe obesity
2. To characterize changes in cardiovascular risk factors (blood pressure, cholesterol, triglycerides, Apo A, Apo B, Apo E, blood glucose, insulin, smoking) over 10 years in medically and surgically treated overweight patients.
3. To study side effects after medical and surgical treatment of obesity
4. To describe changes in drug consumption after medical and surgical treatment of obesity
5. To use the registry study to determine to what extent the validity from the two studied groups may be generalized to all overweight individuals aged 37-57 years with BMI over the given cutoffs (men: BMI≥36, women: BMI≥40)

Ankomstdatum: Dnr 184-90 6/6

**Till forskningsetiska kommittén vid**

Härmed anhålles om prövning av nedan angivna forskningsprojekt som innefattar försök på människa.

Göteborg — Ort
900302 — Datum

Namnteckning (Projektansvarig)

Lars Sjöström, docent, överläkare
Namnförtydligande, titel

Med.klin. I, Sahlgrenska sjukhuset
Institution/klinik

413 45 Göteborg
Adress, tel

Undersökningen har granskats och godkänts av undertecknad(e) för vården ansvarig(a) institutions/klinikchef(er)

Namnteckning(ar)

Per Björntorp, prof.
Namn, titel (klartext)

SOS-sekretariatet, Med.klin. I
Plats för undersökningen

1. Medarbetare (titel, tjänsteinnehav samt arbetsplats) — Multicenterstudie: [x] Ja [ ] Nej

Doc Lars Backman, Kir.klin., Danderyd; Prof. Calle Bengtsson, Allmänmed. inst, Göteborg; Prof. Claude Bouchard, Quebec, Canada; Prof. Sven Dahlgren, Kir.klin., Umeå; Prof. Egon Jonsson, Statens beredning för utvärdering av medicinsk metodik, Stockholm; Doc Bo Larsson, Med.klin. I, SS; Dr Ingmar Näslund, Kir.klin., Örebro; Doc Lars Olbe, Kir.klin., SS; Doc Marianne Sullivan, Psykol. Inst.; Göteborg; Prof. Hans Wedel, Nordiska hälsovårdshögskolan, Göteborg

2. Projekttitel

SOS, Swedish Obese Subjects. En interventionsstudie av fetma (Landsomfattande fas)

3. Försöksobjekt [x] Patient [ ] Frisk försöksperson

Antal: Registerstudie: 8000
Interventionsstudie: 2000 + 2000

4. Beräknad tid för projektets genomförande

14 år

5. Anhållan om tillstånd har insänts till

[ ] Socialstyrelsens läkemedelsavdelning — Datum
[ ] Isotopkommittén vid — Datum
[x] Övrig instans Datainspektionen — Datum 870120

6. Har detta eller liknande projekt godkänts av forskningsetisk kommitté?

[ ] Nej [x] Ja — Datum — Dnr — Kommitte

Pilotstudie om 100 kirurgfall godkänd av berörda etiska kommittéer 1986. Utökning till 300 kirurgfall i pilotstudie godkänd av Göteborg 881216 (152-86), Uppsala 890109 Örebro 881128, Umeå 881213, Stockholm 881221 (KI 86:116), Lund 881214 (427-1988). Linköping som ej varit berörd av pilotstudien, har avstått från att yttra sig.

BESLUT:

Dnr 184-90 - Godkänd 90 06 06 för Göteborgs-regionen.
Ärendet har remitterats till övriga forskningsetiska kommittéer för bedömning.

Godkännande gäller under förutsättning att i förekommande fall tillstånd enligt punkt 5 ovan erhålles.
Det åligger huvudmannen för projektet att rapportera eventuella komplikationer eller biverkningar till institutions/klinikchefen och forskningsetiska kommittén samt beakta eventuellt krav att anmäla läkemedelsprövning till sjukhusets läkemedelskommitté.

Göteborg — Ort
90 06 06 — Datum

Ordförande Carl-Gerhard Gottfries

Sekreterare Nils Svedmyr
