# Supplementary material for: Sex-specific associations between surgery-induced weight loss and cancer outcomes: A post hoc analysis of the prospective, controlled Swedish Obese Subjects study
Source: PLoS Med. 2026 Jan 5;23(1):e1004876. doi: 10.1371/journal.pmed.1004876 (PMC12768343; doi:10.1371/journal.pmed.1004876)
Supplement: S3 Appendix — (PDF) [file pmed.1004876.s004.pdf]

Statistical analysis plan for:

Surgery-induced weight loss and cancer outcomes - Swedish Obese Subjects (SOS) study

The study is registered at ClinicalTrials.gov (NCT01479452)

Principal Investigator: Prof. Lena Carlsson

Analysis plan prepared by Prof. Markku Peltonen

Version 4: 2025-10-01

Contents

1. Study objectives 3
2. Study Design 3
2.1 Overview 3
2.2 Eligibility criteria 3
2.3 Data collection and follow-up 3
3. Study outcomes 4
4. Statistical methods 4
5. Version history 6

1. Study objectives

Surgery-induced weight loss is linked to reduction of cancer risk but less is known about the effect of weight loss on cancer subtypes and which patient subgroups benefit the most. Previously, the Swedish Obese Subjects (SOS) study reported an association between surgery-induced weight loss and lower cancer incidence in women, but not men. The aim of this study was to further examine the link between surgery-induced weight loss and long-term cancer outcomes, focusing on patient subgroups defined by previously identified predictors to optimize treatment benefit. Metabolic changes, particularly glycaemic biomarkers and extent of weight loss, have been suggested as key factors linking bariatric surgery to reduced cancer risk.

The analyses presented here were conceived post hoc and were not specified in the original study protocol. While the Swedish Obese Subjects (SOS) study was designed as a prospective, controlled intervention trial, with overall mortality as primary outcome, the present analyses were developed after trial completion to address additional hypotheses regarding surgery-induced weight loss and cancer risk. Therefore, the analyses will be regarded as explorative.

2. Study Design

2.1 Overview

In the SOS study, 4047 individuals with obesity were enrolled at 25 surgical departments and at 480 primary health care centers between 1 September 1987 and 31 January 2001. A bariatric surgery group of 2007 individuals who underwent surgery was formed, along with a prospectively matched control group of 2040 participants. The study is registered at ClinicalTrials.gov (NCT01479452).

2.2 Eligibility criteria

The inclusion criteria were ages 37 to 60 years and a body-mass index (BMI) of at least 34 in men and 38 in women before or at the time of the matching examination. The exclusion criteria, designed to exclude patients with unacceptable surgical risks, included previous surgeries for gastric or duodenal ulcers, prior bariatric surgery, gastric ulcer in the past 6 months, ongoing or active malignancy within the past 5 years, myocardial infarction in the past 6 months, bulimic eating patterns, drug or alcohol abuse, psychiatric or cooperative problems contraindicating bariatric surgery, other contraindicating conditions (such as chronic glucocorticoid or anti-inflammatory treatment).

2.3 Data collection and follow-up

Baseline examinations took place 4 weeks before the intervention started. Follow-up visits, which included physical examinations and questionnaires, were scheduled at 0.5, 1, 2, 3, 4, 6, 8, 10, 15, and 20 years. Fasting blood samples were collected at baseline and after 2, 10, 15 and 20 years.

Register-based health outcomes will be collected through register-linkage from the Swedish national registries, using unique personal identification numbers in Sweden.

3. Study outcomes

Cancer, death, and emigration data will be obtained by linking SOS participants to national registers. Information on deaths and emigration will be obtained from the Swedish Cause of Death Register and the Swedish Population and Address Register (SPAR).

Information on cancer events will be obtained from The Swedish Cancer Registry, which covers over 95% of malignant tumors, with 99% morphologically verified.

Obesity-related cancers will be defined according to the IARC, and include esophageal adenocarcinoma, postmenopausal breast cancer, cancers of the kidney, colon/rectum, gastric cardia, liver, gallbladder, pancreas, ovary and thyroid, endometrial cancer, multiple myeloma, and meningioma. Other cancers will be classified as non-obesity related. Female-specific cancers include breast, ovarian, endometrial, cervix, and all other gynecological cancers.

All registers are up to date including 31.12.2022.

4. Statistical methods

Baseline characteristics will be compared using t-tests (continuous variables) and Fisher's exact test (dichotomous variables). BMI changes will be analyzed with multilevel linear mixed-effects regression models, separately for men and women. The observations will be considered nested within the individuals, and the confidence intervals (CIs) will be calculated taking the repeated measurements into account.

Patients will be analyzed according to their received intervention (i.e. participants will be included in their original study group until any bariatric surgery happens in the control group or there will be a change in, or removal of, the bariatric surgical procedure in the surgery group).

Participants will be followed until the first occurrence of cancer, death, emigration, or end of register-based follow-up (December 31st, 2022). Those who emigrated, died or remained event-free at the end of follow-up will be treated as censored observations in the analyses. In addition, control-group participants who underwent bariatric surgery during follow-up and surgery-group patients who had a procedure restoring normal anatomy will be censored from the analyses at the time point of these operations.

Kaplan-Meier estimates will be used to compare time to first cancer diagnosis or cancer-related death across treatment groups. Cox proportional hazards models will be used to calculate hazard ratios for surgical treatment effects on overall, obesity-related, and female-specific cancers. The proportional-hazards assumption will be evaluated by assessing the interaction between treatment and the logarithm of time. When the assumption is not fulfilled, Cox regression models will be modified to include time-varying effects. Restricted Mean Survival Time (RMST) approach will be used to compare cancer-free time between the surgery and control groups.

To account for baseline differences between the surgery and control groups, analyses will be adjusted for major cancer risk factors defined by the American Cancer Society, age, sagittal diameter, alcohol consumption, and smoking. In addition, serum insulin levels, which have previously been associated with cancer risk in sub-analyses in the SOS study, will be adjusted for. All covariates are selected a priori the actual analyses and will be included in the models regardless of their actual statistical significance.

To formally test whether baseline insulin modifies the effect of bariatric surgery on cancer risk, an interaction term using the continuous insulin variable will be included in the Cox proportional hazards models. For descriptive purposes, cancer incidence and mortality across subgroups defined by the tertiles of baseline insulin were presented.

The association between weight change and cancer risk will be assessed using the Cox proportional hazard regression model, including one year weight change in the models. These analyses will be restricted to the surgery group only.

In sensitivity analyses, participants with a history of cancer before study inclusion and participants with a cancer event within the first three years after inclusion to the study will be excluded.

5. Version history

Version 4, 2025-10-01 (current version): Additional analyses included during a review process: more detailed analyses on time-varying risk; added analyses with restricted survival-time approach.

Version 3, 2024-11-15: Included analyses on cancer types (obesity-related cancers; female-specific cancers). Analyses on association between weight change and cancer risk in the surgery group added. Register-data was updated to include data until 31.12.2022. Analysis of case-fatality removed.

Version 2, 2023-04-03: Added analyses on cancer mortality, based on reviews of case sheets and autopsy reports. Sensitivity analyses added. Analyses on case-fatality included. More details on statistical methods added.

Version 1, 2021-01-14: First version focusing on time to total cancer incidence (fatal and non-fatal) in the surgery and control groups.
