# Supplementary material for: Sex-specific associations between surgery-induced weight loss and cancer outcomes: A post hoc analysis of the prospective, controlled Swedish Obese Subjects study
Source: PLoS Med. 2026 Jan 5;23(1):e1004876. doi: 10.1371/journal.pmed.1004876 (PMC12768343; doi:10.1371/journal.pmed.1004876)
Supplement: Attachment — Submitted filename: Response to reviewers 2025106_final.docx [file pmed.1004876.s005.docx]

Dear Dr Tosun,

We would like to thank the editorial team and the reviewers for their thorough examination, valuable feedback, and constructive criticism, which have significantly helped to improve the manuscript. Please find our point-by-point responses below.

Comments from the editorial team:

We recommend avoiding the term "female-specific cancers" and using "gynecologic cancers" instead.

**Response:** We appreciate the suggestion. However, we believe that "gynecologic cancers" would not accurately reflect the scope of our analysis, which includes all cancers specifically affecting women, including breast cancer, a major contributor to cancer burden among women but not classified as gynecologic. While a term such as "gynecologic and breast cancers" could be more precise, we are concerned that it may disrupt the flow and readability of the text. To maintain clarity and inclusiveness, we propose retaining the term "female-specific cancers", a term that has also been used in recent studies to encompass both gynecologic and breast cancer (Trias-Llimos et al, Rajitha et al). That said, we are open to further suggestions that balance accuracy with readability.

-Trias-Llimos S, Renteria E, Rutigliano R, Aggarwal A, Moodley J, Unger-Saldana K, et al. Deciphering the sex gap in global life expectancy: the impact of female-specific cancers 1990-2019. J Natl Cancer Inst. 2024;116(12):1934-41.

-Rajitha B, Malla RR, Vadde R, Kasa P, Prasad GLV, Farran B, et al. Horizons of nanotechnology applications in female-specific cancers. Semin Cancer Biol. 2021;69:376-90.

Additionally, in response to Reviewer #1 (minor point #2), we have also clarified in the Abstract and Author summary what is included under the term "female-specific cancers” (lines 52-53).

-----------------------------------------------------------
Comments from the reviewers: 

Reviewer #1: 
This is a well-conducted and timely study leveraging the long-term data from SOS) cohort to explore sex-specific cancer outcomes after bariatric surgery, with a focus on baseline insulin levels. The manuscript is clearly written, the analyses are rigorous, and the findings are both clinically and biologically plausible. The potential for these results to inform risk-stratified preventive oncology strategies is high. However, some  concerns related to interpretation, framing, and generalizability merit attention.

MAJOR POINTS 

1. Clarity on Causality and Stratification: The manuscript occasionally overstates causality or generalizability given the non-randomized design, please clarify throughout the manuscript that the findings reflect associations, not causal inferences. Consider replacing phrases like "surgery reduced cancer risk" with "was associated with a lower cancer risk."

**Response:** Thank you for pointing this out. We have carefully reviewed the manuscript and revised the language to ensure that the findings are consistently presented as associations rather than causal relationships, in line with the non-randomized design.

2. Sex Differences Interpretation; despite the emphasis on sex-surgery interactions, the biological basis remains underexplored. Expand discussion on plausible biological reasons for sex-specific effects (hormonal, adipose distribution, cancer types), or acknowledge this as an open question.

**Response:** Thank you for this suggestion. We have expanded the discussion on possible biological mechanisms behind the sex-specific associations. This section now reads (lines 383-398):

*“Cancer risk in individuals with obesity is multifactorial and influenced by a complex interplay between hormones, the immune system, chronic inflammation, bile acids, and the gut microbiome, which together modulate key signaling pathways [27]. Notably, findings from the prospective, though uncontrolled, LABS study suggest that reductions in insulin, leptin, and ghrelin after bariatric surgery are independently associated with decreased cancer risk [13]. These hormones have all been implicated in tumor biology and may contribute to cancer progression [28-30]. Of note, they also exhibit sex differences in the context of obesity [31, 32], reflecting distinct metabolic profiles that could underlie sex-specific associations between weight loss and cancer risk. In addition, body composition and fat distribution are sexually dimorphic. Men typically accumulate more visceral adipose tissue, whereas women predominantly store subcutaneous fat. These sex-specific patterns of fat distribution are associated with differences in insulin sensitivity, systemic inflammation, and potentially cancer susceptibility [32]. Furthermore, many female-specific cancers, such as breast and endometrial cancer, are estrogen-dependent. Of particular relevance, insulin and estrogen interact in ways that may promote cancer development and progression, including reciprocal receptor activation leading to an increased fraction of bioactive estrogen [27].”*

3. Insulin as a Modifier:  insulin is proposed as a predictor of benefit, only female-specific cancers had significant treatment-insulin interaction p-values. Soften the conclusion that insulin is a "predictor of benefit" across all cancer types. Emphasize where the interaction was statistically robust and where it was only suggestive.

**Response:** Thank you for suggesting this clarification. We have updated the conclusion statements in both the abstract and the discussion to reflect a more cautious interpretation of insulin as a modifier. The revised text now reads (lines 67-69 and 466-469, respectively):

- ***Abstract conclusion****: “Bariatric surgery is associated with a lower risk of cancer and cancer-related mortality in women with obesity, with the strongest association observed for female-specific cancers in women with elevated baseline insulin levels.”*
- ***Discussion conclusion****: “In conclusion, our findings underscore that surgery-induced weight loss is associated with a lower risk of cancer and cancer-related mortality in women with obesity, with the strongest associations observed for female-specific cancers and mortality caused by these cancers in women with high insulin levels.”*

4. Control Group Match:  The matching strategy is not clearly explained in the manuscript body. Include a concise explanation of the matching variables and discuss residual confounding, especially given significant baseline differences.

**Response:** Thank you for the comment, and we apologize that the study inclusion and matching procedures were not sufficiently clear in the original manuscript. We have now clarified the matching strategy in the methods section. Matching between the surgery and control groups was performed at the group level using the method of sequential treatment assignment (Pocock & Simon), based on the following variables: sex, age, smoking, diabetes, weight, height, waist circumference, hip circumference, systolic blood pressure, triglycerides, total cholesterol, menopausal status, current health, monotony avoidance, psychasthenia, quantity and quality of social support, and stressful life events.

Both groups shared identical inclusion criteria and were eligible for surgery. To improve transparency, we have also added the exclusion criteria to the manuscript. These were prior gastric or bariatric surgery, recent or active malignancy, recent myocardial infarction, bulimic eating patterns, substance abuse, psychiatric or cooperative issues contraindicating surgery, and other medical contraindications (e.g., chronic glucocorticoid or anti-inflammatory treatment) (lines 132-145).

***Methods:*** *“Matching of the surgery and control groups was performed at the group level according to the method of sequential treatment assignment [17], based on the following matching variables: sex, age, smoking, diabetes, weight, height, waist circumference, hip circumference, systolic blood pressure, triglycerides, total cholesterol, menopausal status (pre/post), current health, monotony avoidance, psychasthenia, quantity of social support, quality of social support and stressful life events [8]. The surgery and control groups had identical inclusion and exclusion criteria and all participants in both groups were eligible for surgery. Inclusion criteria were age 37–60 years and body mass index (BMI) ≥34 kg/m² for men or ≥38 kg/m² for women. Exclusion criteria were earlier surgery for gastric or duodenal ulcer, earlier bariatric surgery, gastric ulcer in the past 6 months, ongoing malignancy, active malignancy in the past 5 years, myocardial infarction in the past 6 months, bulimic eating pattern, drug or alcohol abuse, psychiatric or cooperative problems contraindicating bariatric surgery, other contraindicating conditions (such as chronic glucocorticoid or anti-inflammatory treatment).”*

We acknowledge that baseline differences remained between the groups, particularly with respect to adiposity and cardiovascular risk factors, which were less favorable in the surgery group (Table 1). These differences are primarily explained by weight changes occurring during the interval between matching and baseline measurements: participants awaiting surgery tended to gain weight, while control subjects tended to lose weight during the waiting period (on average more than one year; Sjöström et al.). This has now been clarified in the results section and added to limitations. Importantly, any bias introduced by this imbalance would likely lead to an underestimation of the benefits of surgery rather than an overestimation (Results, lines 247-249 and 251-255, respectively; Limitations, lines 451-455).

***Results:*** *“Although the two study groups were generally well balanced with respect to baseline characteristics, measures of adiposity and several cardiovascular risk factors were less favorable in the surgery group (****Table 1****).”*

*“These differences are primarily attributable to weight changes that occurred during the interval between matching and baseline measurements: while awaiting bariatric surgery (on average more than one year), participants in the surgery group tended to gain weight, whereas control subjects tended to lose weight [22].”*

***Discussion (limitation):*** *“Second, despite the comprehensive matching strategy, residual confounding cannot be entirely excluded. However, because the surgery group had more adverse baseline risk factors than the controls, any bias introduced by these differences would likely have led to an underestimation, rather than an overestimation, of the benefits of surgery.”*

-Pocock SJ, Simon R. Sequential treatment assignment with balancing for prognostic factors in the controlled clinical trial. Biometrics. 1975;31(1):103-15.

-Sjöström L, Narbro K, Sjöström CD, Karason K, Larsson B, Wedel H, et al. Effects of bariatric surgery on mortality in Swedish Obese Subjects. N Engl J Med. 2007;357(8):741-52.

5. Weight Loss Degree Analysis: Acknowledge the null finding regarding 1 year WL  limitation more explicitly. Discuss whether type of surgery (which affects weight loss and hormonal response) may confound this analysis.

**Response:** Thank you for the comment. We now explicitly acknowledge in the Discussion that our study may have a too limited range of weight loss to detect an association, and that the analyses could have been influenced by the low proportion of SOS patients who underwent gastric bypass (lines 426-431).

***Discussion:*** *“However our study might have too limited range of weight loss within the surgery group and therefore be underpowered to detect an association. Furthermore, the analyses could have been influenced by the low proportion of SOS patients who underwent gastric bypass, a procedure that may induce more pronounced changes in cancer-related metabolic and hormonal risk factors compared with older surgical methods [7, 34].”*

6. Generalizability: most SOS surgical procedures are obsolete. It is worth a concise discussion on extrapolating findings to modern procedures (RYGB, SG) or GLP-1-based pharmacotherapy.

**Response:** Thank you for the comment. We have rephrased the limitations section to clarify that most surgical procedures in the SOS study are no longer commonly performed and that due to the limited number of patients treated with gastric bypass and none with sleeve gastrectomy, procedure-specific cancer risk reduction could not be assessed. Additionally, we acknowledge that the potential cancer-preventive effects of newer pharmacotherapies, such as GLP-1 receptor agonists, remain to be evaluated in future studies (lines 455-462).

*“…because the SOS study was initiated in the 1980s, most participants underwent surgical procedures that are no longer commonly performed. As a result, the number of patients treated with gastric bypass was limited, and none underwent sleeve gastrectomy. This restricts our ability to assess modern procedure-specific effects on cancer risk. Moreover, the potential cancer-preventive effects of newer obesity treatments, including GLP-1 receptor agonists, remains to be determined. Thus, long-term studies involving contemporary surgical techniques and pharmacotherapies are needed to evaluate the generalizability of our findings.”*

MINOR ISSUES:

1. Consider adding risk tables below Kaplan-Meier curves for clarity.         

**Response:** Thank you for the suggestion. We agree that risk tables enhance the interpretability of Kaplan-Meier curves but would like to note that risk tables are already included below the Kaplan-Meier curves in the current version of the manuscript.

2. Define "female-specific cancers"  in the Abstract and Key Points.

Response: Female-specific cancers are now defined as “breast and gynecologic cancers” in the Abstract and Author summary (lines 52-53 and 83-84, respectively.

3. Emphasize again in the Discussion that cancer was not a predefined endpoint

**Response:** We had already addressed this point in the limitations section but have now clarified the wording to ensure it is more explicit. If the reviewer’s suggestion is that this aspect should be introduced earlier in the discussion, we are willing to make further adjustments accordingly (lines 450-452).

***Limitation:*** *“…when the SOS study was initiated, obesity had not yet been linked to increased cancer risk. Consequently, cancer incidence was not a predefined endpoint, and all related analyses are exploratory.”*

Reviewer #2: The Swedish Obese Subjects (SOS) prospective cohort study recruited weight loss surgery patients and matched controls, and previously published analyses of this cohort found reduced cancer incidence in women but not in men. This study presents similar analyses based on the same cohort with 17 additional years of follow up and considering additional predictors. The study confirmed earlier findings of reduced cancer incidence and mortality in women but not in men, with the effect further mediated by baseline insulin level. 
Overall, this manuscript is an excellent initial submission. It is clearly written, logically presented, and cogently argued. That said, there are some areas of methodological concerns and opportunities to improve clarity. I'd also encourage the authors to complete and include a STROBE checklist (<https://www.equator-network.org/reporting-guidelines/strobe/>) with their next submission.

**Response:** We have now included a STROBE checklist as a supplementary file (S1 Checklist).

Detailed feedback on the manuscript is provided below. My primary focus has been methodological, but I have made a few broader points as well.  All items are major unless indicated otherwise.
1.      The Introduction is both comprehensive and succinct. Very well done.

**Response:** Thank you

2.      [MINOR] Consider presenting the proportion of causes of death where the official and study causes differed.

**Response:** We did consider this at the submission stage. Notably, the majority (>97%) of study-defined cancer-related deaths were also attributed to cancer in the Cause of Death Register, supporting the consistency of our classification. We therefore did not believe that a direct comparison of study-defined versus official causes would meaningfully add to the manuscript, but we have added a clarifying comment in the Data Collection section. This section now reads (lines 182-187):

“*Causes of death were also independently reviewed by two authors using case sheets and autopsy reports. If the cause of death determined in the study diverged from the official record, the study-determined direct cause was used, because the official cause of death in some cases reflects the underlying preventable cause (e.g., obesity) in the chain of events leading to death. However, a majority (>97%) of study-defined cancer-related deaths were also officially attributed to cancer in the Swedish Cause of Death register.*”

3.      Generally, p-values should not be used in descriptive tables (see section 14a of the expanded STROBE guidelines <https://journals.plos.org/plosmedicine/article?id=10.1371/journal.pmed.0040297)>. Suggest the authors remove from Methods and Table 1.

**Response:** Thank you for the suggestion. We have removed p-values from Table 1 and the corresponding description in the Methods section, in accordance with the STROBE guidelines.

4.      I was pleased to see the authors considering missingness in their methods but did want to flag that missing at random (MAR) is a relatively strong and untestable assumption. I suggest the authors provide further justification, including any predictors of missingness the authors considered and whether they were included in the models.

**Response:** Thank you for this comment. First, it should be noted that in this manuscript, the MAR assumption mainly refers to the analysis of changes in body weight, presented in Supplementary figure 2. All other analyses are based on nearly complete baseline data in the two treatment groups, and on longitudinal follow-up from national health registers (e.g., the Cause of Death Register, the Swedish Cancer Registry, the Swedish Population and Address Register) using a time-to-event analysis approach. As the coverage of Swedish national registers approach 100%, we have assumed that the coverage is complete.

Missing data on body weight during follow-up, captured through individual health examinations within the SOS study, have been analyzed in previous SOS publications focusing on treatment effects on body weight (e.g., Sjöström et al, NEJM 2004, Table 1; Carlsson et al, NEJM 2012; S-Table 4; Sjöström et al, JAMA 2014; eTable 2). A number of factors are associated with drop-out. For example, the likelihood of non-participation in health examinations was higher in the control group than in the surgery group. Similarly, the likelihood of non-participation was also higher in men than in women.

However, because our main analyses in this manuscript do not depend on examination-based data during the follow-up, we would prefer to not expand the manuscript on this topic.

-Sjöström L, Lindroos AK, Peltonen M, Torgerson J, Bouchard C, Carlsson B, et al. Lifestyle, diabetes, and cardiovascular risk factors 10 years after bariatric surgery. N Engl J Med. 2004;351(26):2683-93.

-Bariatric surgery and prevention of type 2 diabetes in Swedish obese subjects. NEJM. 2012;367(8):695-704.

-Sjöström L, Peltonen M, Jacobson P, Ahlin S, Andersson-Assarsson J, Anveden A, et al. Association of bariatric surgery with long-term remission of type 2 diabetes and with microvascular and macrovascular complications. JAMA. 2014;311(22):2297-304.

5.      The descriptions of the statistical modelling approaches in the Methods are a good start, but more detail is needed. If modelling results are presented, then enough detail should be included in the Methods so an interested reader can replicate the analyses. Specifically, while the authors have noted the model type they have used (e.g., "multilevel mixed-effect (linear) regression" and Cox PH), it's important to clearly state the variables in the models (fixed and random effects for hierarchical models, cluster variables for GEE etc.). Including abridged modelling code as a supplement can also help when more complex analyses are being undertaken.

**Response**: We have clarified the model specifications. This part of the Statistical methods section now reads (lines 196-201):

*“BMI changes were analyzed using multilevel linear mixed-effects regression models assuming normally distributed errors, separately for men and women. The observations were considered nested within the individuals, and the confidence intervals (CIs) were calculated taking the repeated measurements into account. Treatment, time and their interaction as well as age were considered fixed, and the individual patients were considered random-effects.”*

6.      Relatedly, I found the manuscript a little muddled when it came to stratified analyses vs. analyses controlling for variables (with or without interactions). Suggest the authors make this clearer in their Methods and Results.

**Response:** We have rewritten the section on the adjusted analyses in the Methods. Please see also response under point 7 below. All analyses were adjusted, including the stratified analyses (lines 220-230).

**Statistical Methods:** *“To account for baseline differences between the surgery and control groups, analyses were adjusted for major cancer risk factors defined by the American Cancer Society; age, sagittal diameter (a proxy for intra-abdominal adiposity), alcohol consumption, and smoking, [20] as well as serum insulin levels, which have previously been associated with cancer risk in sub-analyses in the SOS study [14, 15], as well as in the LABS study [13]. All covariates were selected a priori the actual analyses and included in the models regardless of their actual statistical significance.*

*To formally test whether baseline insulin modified the effect of bariatric surgery on cancer incidence and cancer-related mortality, we included an interaction term using the continuous insulin variable in Cox proportional hazards models. These analyses were also adjusted for other risk factors as described above.”*

7.      Consider explaining how variables included in the statistical models were chosen e.g., prior research, expert knowledge, DAG.

**Response:** Most covariates (age, sagittal diameter, alcohol consumption, and smoking status) were selected based on established cancer risk factors listed by the American Cancer Society. Serum insulin was included due to prior findings in the Swedish Obese Subjects (SOS) study, where hyperinsulinemia was associated with increased cancer risk and findings reported from the LABS study. This is now explained in the Methods (lines 220-226):

*“To account for baseline differences between the surgery and control groups, analyses were adjusted for major cancer risk factors defined by the American Cancer Society; age, sagittal diameter (a proxy for intra-abdominal adiposity), alcohol consumption, and smoking, [20] as well as serum insulin levels, which have previously been associated with cancer risk in sub-analyses in the SOS study [14, 15], as well as in the LABS study [13]. All covariates were selected a priori the actual analyses and included in the models regardless of their actual statistical significance.”*

8.      I am not at all clear how case-fatality rates were calculated and, in the Results, why no statistics are presented. Suggest the authors clarify methods and present analytical results (or justify their absence).

**Response:** We thank the reviewer for pointing this out. The methods for case-fatality rates were a remnant from an earlier draft and did not add meaningful information to the manuscript. We have therefore removed the text entirely from the Methods.

9.      [MINOR] I found the sentence in the Statistical Analysis section starting "Cancer incidence and cancer mortality were tracked…" a little hard to read. Consider rewording as being clear on events and censoring criteria is crucial.

**Response:** Thank you for pointing this out. The text has been revised for clarity and now reads (lines 206-211):

“*Participants were followed until the first occurrence of cancer, death, emigration, or end of register-based follow-up (December 31st, 2022), whichever came first. Participants who emigrated, died or remained event-free at the end of follow-up were treated as censored observations in the analyses. In addition, control-group participants who underwent bariatric surgery during follow-up and surgery-group patients who had a procedure restoring normal anatomy were also censored from the analyses at the time of these operations.”*

10.     While hazard ratios are the generally accepted statistic for time-to-event analyses, they have some well-recognised limitations (see <https://doi.org/10.1093/ehjacc/zuae017> and <https://evidence.nejm.org/doi/10.1056/EVIDe2300142)>. The authors may wish to consider supplementing key HR outcomes with another statistic like difference in median survival or restricted mean survival time to assist with interpretability.

**Response:** Thank you for this helpful suggestion. We have expanded the manuscript to include differences in restricted mean survival times alongside hazard ratios. The following has been added to the Statistical Methods section (lines 217-218 and 237-238, respectively):

*“In addition, a Restricted Mean Survival Time (RMST) approach was used to compare cancer-free time between the surgery and control groups.”*

*“RMSTs were analyzed with the Stata strmst2-package.[21]”*

The Results section has been updated accordingly (lines 268-270):

*”Over 31.5 years of follow-up, the difference in restricted mean cancer-free time between the surgery and control groups was 1.4 years [95% CI: 0.8 to 2.1], p<0.001.”*

11.     Given the long follow-up, I suggest the authors check that the proportional hazard (PH) assumption remains valid when using Cox regression (e.g., Schoenfeld residuals). If PH is violated, consider adjusting the Cox model (e.g., including time-varying covariates) or using alternative approaches that do not assume proportional hazards (e.g., RMST).

**Response:** We tested the proportional hazards assumption and found no evidence of violation for cancer incidence in men or for cancer mortality in either sex. For cancer incidence in women, however, the association between surgery and treatment was more pronounced in the early follow-up period as compared to the later years. We have now included additional analyses and results to reflect this.

In the **Methods**, we now state (lines 215-217):

*“The proportional-hazards assumption was evaluated by assessing the interaction between treatment and the logarithm of time. When the assumption was not met, Cox regression models were modified to include time-varying effects.”*

In the **Results**, the following two sentences have been added (lines 272-274 and 281-282, respectively):

*”* *The association between surgery and cancer incidence in women was time-dependent, being more pronounced early during follow-up.”*

*“The association between surgery and cancer incidence differed by sex (adjusted p for sex-treatment interaction=0.013; accounting for non-proportional hazard p=0.011).”*

12.     It's not clear to me how the authors handled patients with a history of cancer before inclusion into the study nor patients who experienced a cancer event in the first three years. I see from the methods the authors conducted sensitivity analyses excluding "… cancer events within the first three years and pre-inclusion events." Does this mean in the main analyses patients with a history of cancer are contributing time as if they were cancer free? In the sensitivity analyses was the event dropped but the patient kept, or was the patient dropped entirely. I suggest the authors make their methods clearer here and carefully consider any risk of bias arising from their choices.

**Response:** We apologize for the unclear description of sensitivity analyses. In the main analyses, all participants in the SOS study (i.e. N=4,047), including those with a history of cancer before study inclusion, were included in the analyses and contributed follow-up time. In the sensitivity analyses, however, participants with a history of cancer, or who experienced a cancer event during the first three years after inclusion, were excluded entirely from the analyses (i.e. the participant was removed, not only the event).

We have clarified this in the Methods, which now reads (lines 233-235):

*“In sensitivity analyses, participants with a history of cancer before study inclusion were excluded. In addition, to account for undiagnosed baseline cancer, participants with a cancer event within the first three years after inclusion were also excluded from the analyses.”*

13.     When presenting results from a Cox regression (e.g., Fig 1), if a p-value is to be presented in addition the to the HR and CI then the p-value should come from the Cox model, not a log-rank test on the same data. The log-rank test can be used in a simpler, unadjusted analyses but mixing a log-rank test p-value with Cox regression results is confusing at best and misleading at worst.

**Response:** As suggested, we have removed the log-rank p-values from the figures to avoid confusion and now present only the hazard ratios, confidence intervals, and corresponding p-values from the Cox regression models.

14.     The authors present many p-values and make multiple references to "(statistical) significance". This is risky because the more claims of significance are made, the greater the risk of false positives and the more important it becomes to consider multiple testing correction strategies like the Bonferroni adjustment. Assuming the authors would prefer to avoid this, I'd suggest excluding p-values, instead just presenting point estimates and 95% CIs. I'd also suggest moderating or changing language away from statistical significance as an all-or-nothing threshold. One option is to talk in terms of strength of evidence e.g., "strong/moderate/some evidence of…" or "no evidence was found of…".

**Response:** Thank you for your helpful comment. In accordance with the journal’s policy, we are required to report both p-values and 95% confidence intervals for the main results. However, we have revised the manuscript to better reflect the strength of evidence rather than relying solely on binary interpretations of statistical significance. We hope these revisions address your concerns while remaining compliant with the journal’s guidelines. In addition, the study is now clearly identified as a post-hoc analysis, and therefore all the findings should be regarded as explorative.

15.     While historically categorising continuous variables has been common practice, recent methodological research discourages this ( <https://www.bmj.com/content/332/7549/1080.1> ). Suggest the authors consider analysing continuous variables as-is, including insulin and 1-year weight loss.

**Response:** While the results are presented in tertiles for ease of interpretation and visualization, all interaction analyses were conducted using the underlying continuous variable. This approach preserves statistical power and avoids arbitrary categorization, ensuring that the interaction effects are modeled more accurately. The tertile-based presentation is intended to aid readers in understanding the distribution and practical implications of the findings, without compromising the rigor of the statistical methodology.

This has also been clarified in methods (lines 227-232):

*"To formally test whether baseline insulin modified the effect of bariatric surgery on cancer incidence and cancer-related mortality, we included an interaction term using the continuous insulin variable in Cox proportional hazards models. These analyses were also adjusted for other risk factors as described above. For descriptive purposes, cancer incidence and mortality across subgroups defined by the tertiles of baseline insulin were presented in the figures."*

16.     "After extending the follow-up period by another 17 years, we now observe a 22% reduction in post-surgery cancer risk among women…" This is not correct, hazard ratios and risk/probabilities are not interchangeable because hazard ratios reflect relative rates over time rather than absolute differences in risk. Suggest rewording here and anywhere else hazard ratios are interpreted as relative risk.

**Response:** We have revised the wording throughout the manuscript accordingly. Two examples are given below (lines 362-365 and 365-368, respectively):

*"After extending the follow-up period by another 17 years, surgery was associated with a 22% lower hazard (HRadj=0.78) of incident cancer among women, with a strong indication of sex differences in treatment outcomes, aligning with associations reported in earlier retrospective studies [6, 9, 12]."*

*"Our results also show that surgery was associated with a 22% lower hazard (HRadj=0.78) of cancer mortality among women, while no clear association was observed in men, consistent with retrospective findings from the Utah Population Database [6]."*

17.     I think there are two important limitations to this study that were not identified by the authors. Patient characteristics like smoking and alcohol consumption are important cancer risk factors that may be susceptible to self-report bias. Given the larger proportion of women in the study, the study may be underpowered to detect treatment effects in men. Suggest the authors consider these for inclusion.

**Response:** We agree that self-reported measures such as smoking and alcohol consumption may be subject to bias. However, the use of a matched control group helps mitigate this concern, as any potential misclassification is likely to be non-differential between groups. These variables were also included as covariates in the adjusted analyses to further reduce confounding.

Regarding the concern about statistical power in men, we acknowledge the smaller sample size in this subgroup and have added this as a limitation in the Discussion (lines 463-465). Still, the long follow-up of the SOS study partly mitigates this.

*”Finally, the number of men in the SOS study is considerably lower than the number of women, affecting the sex-specific analyses.”*

18.     Aside from the few items above, the Discussion is well-constructed, clearly follows from the presented results, and is convincingly argued. Great work! 

**Response:**  Thank you!

Reviewer #3: Dear Dr. Taube and colleagues,

This is an interesting work, studying the very long term medical outcomes of bariatric surgery on cancer. I find the results surprising and interesting, in particular the sex dependent effects of the surgery and effects on female cancers. The effects size are meaningful, the results statistically signifnicant, and I believe they will be of interest to the bariatric and surgical community. As for the cancer community, it is hard to say, there are many factors affecting cancer in such a long term followup. 

1. It wasn't entirely clear to me why the specific cutoffs for insulin were chosen, or why look at insulin specifically. I was wondering if there was any interaction between incidence of T2D, or fasting glycemia before or during the long followup (if available) and the incidence of cancer, as a mediating factor.

**Response:** We apologize that the rationale behind the insulin-stratified analyses was not sufficiently clear in the original manuscript. We have now clarified this in the introduction (lines 117-120):

*“Moreover, metabolic changes following bariatric surgery were recently proposed as independent predictors of reduced cancer risk, with glycemic biomarkers showing the strongest associations [13]. This is in line with previous sub-analyses of the SOS cohort which have indicated that insulin may be a modifier of bariatric surgery benefit [14, 15].”*

Our decision to focus on insulin was guided by its central role in obesity-related metabolic dysregulation and prior evidence linking hyperinsulinemia to cancer risk and to differential associations between bariatric surgery and cancer-related outcomes in the SOS study. While the results are presented in tertiles for ease of interpretation and visualization, all interaction analyses were conducted using insulin as a continuous variable. This approach preserves statistical power and avoids arbitrary categorization, ensuring that the interaction effects are modeled more accurately. The tertile-based presentation reflects the actual distribution of insulin in our study population, facilitates balanced group sizes, and helps illustrate potential dose–response relationships in a way that is accessible to readers, without compromising the rigor of the statistical methodology.

We agree that the proposal to evaluate an interaction between incidence of T2D and the incidence of cancer is interesting, but unfortunately the SOS study is not ideal to do this. Sweden does not have a register for this long time-period, starting from the 1980s, where the incidence of diabetes could be reliably identified. We do have information about diabetes incidence from the individual health examinations within the SOS protocol, with measurements on blood glucose and HbA1c, as well as self-reported diabetes mediation. The problem with this data is that these examinations were conducted only at baseline, and after 2, 10, 15 and 20 years of follow-up. Consequently, the time point for incident diabetes is only approximate. While it might be possible to account for this with statistical methods, we do feel this would be outside of the scope of the present manuscript.

2. I was also curious if there was an age dependent or menopause dependent effects on the outcome, obesity is considered a risk factor for breast cancer at an older age and less so (even protective?) at a younger age. Can the authors look at any interactions?

**Response:** Thank you for your thoughtful comment. We did explore potential interactions between obesity and menopausal status in our initial analyses. However, we found no evidence of an interaction. Given the already substantial scope of the manuscript, we chose not to include these negative findings in the main text.

Moreover, emerging evidence suggests that obesity shifts the timing of cancer appearance to individuals of younger age (Berger) meaning that the relationship between obesity and breast cancer risk may be less dependent on menopausal status than previously thought. Indeed, recent retrospective reports on bariatric surgery in women with obesity showed a reduced risk of both premenopausal and postmenopausal breast cancer, compared to women not undergoing surgery. (Feigelson et al, Adams et al).

-Berger NA. Young Adult Cancer: Influence of the Obesity Pandemic. Obesity (Silver Spring). 2018;26(3):443-450. doi:10.1002/oby.22137

-Feigelson HS, Caan B, Weinmann S, Leonard AC, Powers JD, Yenumula PR, et al. Bariatric surgery is associated with reduced risk of breast cancer in both premenopausal and postmenopausal women. Ann Surg. 2020;272(6):1053-1059.

-Adams TD, Meeks H, Fraser A, Davidson LE, Holmen J, Newman M, et al. Long-term cancer outcomes after bariatric surgery. Obesity (Silver Spring). 2023;31(9):2386-2397.

3.The authors point well to the stregnths and weaknesses of the study. 
Congratulations on this long followup and intersting study

**Response:** Thank you!

Any attachments provided with reviews can be seen via the following link: 
--------------------------------------------------------- ---
General editorial requests:
(Note: not all will apply to your paper, but please check each item carefully)

*       We ask every co-author listed on the manuscript to fill in a contributing author statement, making sure to declare all competing interests. If any of the co-authors have not filled in the statement, we will remind them to do so when the paper is revised. If all statements are not completed in a timely fashion this could hold up the re-review process. If new competing interests are declared later in the revision process, this may also hold up the submission. Should there be a problem getting one of your co-authors to fill in a statement we will be in contact. Please do not add or remove authors without first discussing this with the handling editor. You can see our competing interests policy here: <http://journals.plos.org/plosmedicine/s/competing-interests>.

**Response:** NA

*       Please upload any figures associated with your paper as individual TIF or EPS files with 300dpi resolution at resubmission; please read our figure guidelines for more information on our requirements: <http://journals.plos.org/plosmedicine/s/figures>. While revising your submission, we strongly recommend that you use PLOS's NAAS tool (<https://ngplosjournals.pagemajik.ai/artanalysis>) to test your figure files. NAAS can convert your figure files to the TIFF file type and meet basic requirements (such as print size, resolution), or provide you with a report on issues that do not meet our requirements and that NAAS cannot fix.

**Response:** Done

After uploading your figures to PLOS's NAAS tool - <https://ngplosjournals.pagemajik.ai/artanalysis>, NAAS will process the files provided and display the results in the "Uploaded Files" section of the page as the processing is complete.
If the uploaded figures meet our requirements (or NAAS is able to fix the files to meet our requirements), the figure will be marked as "fixed" above. If NAAS is unable to fix the files, a red "failed" label will appear above.
When NAAS has confirmed that the figure files meet our requirements, please download the file via the download option, and include these NAAS processed figure files when submitting your revised manuscript.

**Response:** Done

*       Please ensure that the paper adheres to the PLOS Data Availability Policy (see <http://journals.plos.org/plosmedicine/s/data-availability)>, which requires that all data underlying the study's findings be provided in a repository or as Supporting Information. For data residing with a third party, authors are required to provide instructions with contact information (web or email address) for obtaining the data. Please note that a study author cannot be the contact person for the data. PLOS journals do not allow statements supported by "data not shown" or "unpublished results." For such statements, authors must provide supporting data or cite public sources that include it.

**Response:** The data are subject to legal restrictions under national legislation. In accordance with the PLOS Data Availability Policy, we have included a Data Availability Statement that provides instructions and appropriate contact information for obtaining access to the data (lines 493-501).

*       We expect all researchers with submissions to PLOS in which author-generated code underpins the findings in the manuscript to make all author-generated code available without restrictions upon publication of the work. In cases where code is central to the manuscript, we may require the code to be made available as a condition of publication. Authors are responsible for ensuring that the code is reusable and well documented. Please make any custom code available, either as part of your data deposition or as a supplementary file. Please add a sentence to your data availability statement regarding any code used in the study, e.g. "The code used in the analysis is available from Github [URL] and archived in Zenodo [DOI link]" Please review our guidelines at <https://journals.plos.org/plosmedicine/s/materials-software-and-code-sharing> and ensure that your code is shared in a way that follows best practice and facilitates reproducibility and reuse. Because Github depositions can be readily changed or deleted, we encourage you to make a permanent DOI'd copy (e.g. in Zenodo) and provide the URL. 

**Response:** NA

*       ETHICS STATEMENTS: Please also include an appropriate contact (web or email address) for data inquiries. Please note that this cannot be a study author.

**Response:** A contact address for data inquiries has been added to the Data Availability Statement (line 501).

FORMATTING - GENERAL
*       Abstract: Please structure your abstract using the PLOS Medicine headings (Background, Methods and Findings, Conclusions). Please combine the Methods and Findings sections into one section.

**Response:** Done

*       At this stage, we ask that you include a short, non-technical Author Summary of your research to make findings accessible to a wide audience that includes both scientists and non-scientists. The Author Summary should immediately follow the Abstract in your revised manuscript. This text is subject to editorial change and should be distinct from the scientific abstract. Ideally each sub-heading should contain 2-3 single sentence, concise bullet points containing the most salient points from your study. In the final bullet point of 'What Do These Findings Mean?', please include the main limitations of the study in non-technical language. Please see our author guidelines for more information: <https://journals.plos.org/plosmedicine/s/revising-your-manuscript#loc-author-summary>.

**Response:** Done

*       Please express the main results with 95% CIs as well as p values. When reporting p values please report as p<0.001 and where higher as the exact p value p=0.002, for example. Throughout, suggest reporting statistical information as follows to improve clarity for the reader "22% (95% CI [13%,28%]; p</=)". Please be sure to define all numerical values at first use.

**Response:** Done

*       Please include page numbers and line numbers in the manuscript file. Use continuous line numbers (do not restart the numbering on each page). 

**Response:** Done

*       Please cite the reference numbers in square brackets. Citations should precede punctuation.

**Response:** Done

FIGURES AND TABLES
*       Please provide titles and legends for all figures and tables (including those in Supporting Information files).

**Response:** Done

*       Please define all abbreviations used in each figure/table (including those in Supporting Information files).

**Response:** Done

*       Please consider avoiding the use of red and green in order to make your figure more accessible to those with color blindness.

**Response:** NA

SUPPLEMENTARY MATERIAL
*       Please note that supplementary material will be posted as supplied by the authors. Therefore, please amend it according to the relevant comments outlined here.

*       Please cite your Supporting Information as outlined here: <https://journals.plos.org/plosmedicine/s/supporting-information>

**Response:** Done

REFERENCES
*       PLOS uses the numbered citation (citation-sequence) method and first six authors, et al.

*       Please ensure that journal name abbreviations match those found in the National Center for Biotechnology Information (NCBI) databases (<http://www.ncbi.nlm.nih.gov/nlmcatalog/journals>), and are appropriately formatted and capitalised.

**Response:** Done

*       Where website addresses are cited, please include the complete URL and specify the date of access (e.g. [accessed: 12/06/2025]).

**Response:** NA

*       Please also see <https://journals.plos.org/plosmedicine/s/submission-guidelines#loc-references> for further details on reference formatting.

**Response:** Done

STUDY TYPE-SPECIFIC REQUESTS 
*       Abstract: Please include the study design, population and setting, number of participants, years during which the study took place (enrollment and follow up), length of follow up, and main outcome measures.

**Response:** Done (see below).

*       Please be explicit in the title and the abstract that is a post-hoc sub-study/follow-on study of the primary trial and confirm that the current study was not a pre-specified longterm outcome of the primary trial. We suggest reporting in-line with CONSORT explicitly stating the sub-study nature and ensuring that the abstract details the main trial items in 2-3 sentences, including the study population, dates, intervention and primary outcome. The majority of the abstract should then describe the complete details of this post-hoc sub-study.

**Response:** We have now made the nature of the study explicit in both the title and the abstract. The title has been revised to: *“A post hoc analysis of sex-specific associations between baseline insulin levels and cancer outcomes after surgery-induced weight loss: Findings from the prospective, controlled Swedish Obese Subjects study”*.

The abstract has been updated to clearly state that this is a post-hoc analysis of the Swedish Obese Subjects (SOS) study and that the current analysis was not a pre-specified long-term outcome of the primary trial. In accordance with CONSORT guidelines for reporting secondary analyses, the abstract now includes a summary of the main trial, covering the study population, dates, intervention, and primary outcome.

*       Please comment on whether the current study received ethical approval that is different from that of the primary trial.

**Response:** The original Swedish Obese Subjects (SOS) study received ethical approval from the relevant institutional review boards, and all participants provided informed consent for future analyses of collected data (approval number 184-90, date of decision June 6, 1990; renewed approval number T508-17, date of decision June 12, 2017). Approval for linkage with the national cancer register was obtained in 2001 (reference number S604-01, approved June 16, 2002). This is described in the methods (lines 396-401).

*       Please ensure that the study is reported according to the STROBE (or appropriate STOBE extension) guideline (available from: <https://www.equator-network.org/reporting-guidelines/strobe)> and include the completed STROBE (or STROBE extension) checklist as Supporting Information. Please add the following statement, or similar, to the Methods: "This study is reported as per the Strengthening the Reporting of Observational Studies in Epidemiology (STROBE) guideline (S1 Checklist)." When completing the checklist, please use section and paragraph numbers, rather than page numbers. 

**Response:** Done

*       For all observational studies, in the manuscript text, please indicate: (1) the specific hypotheses you intended to test, (2) the analytical methods by which you planned to test them, (3) the analyses you actually performed, and (4) when reported analyses differ from those that were planned, transparent explanations for differences that affect the reliability of the study's results. If a reported analysis was performed based on an interesting but unanticipated pattern in the data, please be clear that the analysis was data driven. 

**Response:** These elements are already included in the manuscript. No data-driven analyses are included.

*       Please state in the Methods section whether the study had a prospective protocol or analysis plan. If a prospective analysis plan (from your funding proposal, IRB or other ethics committee submission, study protocol, or other planning document written before analyzing the data) was used in designing the study, please include the relevant document(s) with your revised manuscript as a Supporting Information file to be published alongside your study and cite it in the Methods section. A legend for this file should be included at the end of your manuscript. If no such document exists, please make sure that the Methods section transparently describes when analyses were planned, and when/why any data-driven changes to analyses took place. Changes in the analysis, including those made in response to peer review comments, should be identified as such in the Methods section of the paper, with rationale.

**Response:** The statistical analysis plan is now included as a supporting information file and the following text has been added to the methods section (lines 164-170):

*“The analyses presented here were conceived post hoc and were not specified in the original trial protocol or analysis plan (****S2 Appendix****). While the Swedish Obese Subjects (SOS) study was designed as a prospective, controlled intervention trial, the present analyses were developed after trial completion of the analyses on primary outcome, to address additional hypotheses regarding surgery-induced weight loss and cancer risk. These analyses were prespecified, and a statistical analysis plan was prepared before undertaking them (****S3 Appendix)****.”*

*       We noticed that you used "data not shown" in the manuscript. We do not allow these references, as the PLOS data access policy requires that all data be either published with the manuscript or made available in a publicly accessible database. Please amend the supplementary material to include the referenced data or remove the references."

**Response:** The case-fatality rates were a remnant from an earlier draft and did not add meaningful information to the manuscript. We have therefore removed them entirely from the Results.

*       We have noticed that you have uploaded Supporting Information files, but you have not included a list of legends. Please add a full list of legends for your Supporting Information files after the references list."

**Response:** Done (lines 682-700).
