# Supplementary material for: Sex-specific associations between surgery-induced weight loss and cancer outcomes: A post hoc analysis of the prospective, controlled Swedish Obese Subjects study
Source: PLoS Med. 2026 Jan 5;23(1):e1004876. doi: 10.1371/journal.pmed.1004876 (PMC12768343; doi:10.1371/journal.pmed.1004876)
Supplement: Attachment — Submitted filename: Reviewer_comments251209_final.docx [file pmed.1004876.s006.docx]

Dear Dr. Tosun,

We thank you and the editorial team for the constructive feedback provided, and we are pleased that the revisions were largely considered satisfactory. We have carefully addressed the remaining points raised in this round of review, including the statistical concerns and editorial requests. Below, we provide a detailed, point-by-point response outlining how each comment has been incorporated into the revised manuscript.

Reviewer #2: I thank the authors for their considered and comprehensive responses to my comments and note the manuscript is reading very well. In all but three cases, I am happy to fully accept the authors' proposed updates and consider the items finalised. However, I do suggest the authors revisit the three items below (numbers reference my original review items). In any cases where I have suggested wording, please treat this as illustrative only i.e., I am not dictating the only acceptable wording.

3. The authors have removed the p-values from the Baseline Characteristics table as requested but have not yet updated the Methods to match. Suggest removing the first sentence of the Statistical Analysis section (line 195).

**Response:** Thank you for pointing this out. The sentence has been removed.

4. [MINOR] I take the authors' point on missingness but worry a reader may have the same concerns I did with the text as it stands on first read. Perhaps even something as simple as 'Due to the nature of the available data, data missingness was expected to be negligible in the primary outcomes and exposures. Where data were missing, Missing data were assumed to be "missing at random"…' This is just a wording tweak and is not required for me to recommend acceptance if the authors feel strongly.

**Response:** The section has been modified and now reads (lines 315-317): “*Due to the nature of the available data, missingness was expected to be negligible for the primary outcomes and exposures. Where data were missing, we assumed that values were missing at random, and all available observed data were included in the analyses.*”

12. Thanks to the authors for clarifying that patients in the in the main analyses were not affected by the issues raised in this point. Unfortunately, the sensitivity analysis as specified is at risk of bias. See [https://link.springer.com/article/10.1007/s12561-024-09442-9](https://link.springer.com/article/10.1007/s12561-024-09442-9) for full methodological details, but intuitively the problem can be considered as one of proportional hazards: Cox regression assumes proportional hazards but the approach as specified forces a much less hazardous first three years than follow-up afterwards. There are several ways to address this, the simplest being to include all participants whether they have an event in the first three years or not and "start the clock" at three years for all patients. That is, remove the first 3 years of person-time and all events occurring then for all participants, not just those who had early events.

**Response:** Thank you for this suggestion, we have modified the sensitivity analyses as suggested. The Statistical methods section now reads (lines 356-358):

*“In sensitivity time-to-event analyses accounting for undiagnosed baseline cancer, follow-up time for all participants was initiated at year 3 after baseline, and only cancer events occurring after this initial 3-year follow-up period were included.“*

And the corresponding text in the Results now reads (lines 406-408):

*“Sensitivity analyses produced similar estimates when follow-up was started at year 3 after (HRadj=0.82; 95% CI [0.70, 0.95]; p=0.010) (****sFigure 3 in S1 Appendix****), ….”*

------------------------------------------------------------
Requests from Editors:

GENERAL
* Please confirm that your title complies with to PLOS Medicine's style. Your title must be nondeclarative and not a question. It should begin with main concept if possible. "Effect of" should be used only if causality can be inferred, i.e., for an RCT. Please place the study design ("A randomized controlled trial," "A retrospective study," "A modelling study," etc.) in the subtitle (ie, after a colon).

Editorial note: Based on the Abstract, we don’t think ‘baseline insulin levels’ should be in the title. We suggest: “Sex-specific associations of cancer outcomes after surgery-induced weight loss: A post hoc analysis of the Swedish Obese Subjects study"

**Response:** We revised the title but chose to retain “prospective, controlled” in the subtitle to accurately reflect the study design and remain consistent with PLOS Medicine style. The amended title now reads (lines 1-3): *“Sex-specific associations between surgery-induced weight loss and cancer outcomes: A post hoc analysis of the prospective, controlled Swedish Obese Subjects study”*

* Statistical reporting: Please revise throughout the manuscript, including tables and figures.
- Please report statistical information as follows to improve clarity for the reader ""22% (95% CI [13,28]; p</=)"".
- Please separate upper and lower bounds with commas instead of hyphens as the latter can be confused with reporting of negative values.
- Please repeat statistical definitions (HR, CI etc.) for each set of parentheses.

**Response:** Done

* Please ensure that all abbreviations are defined at first use throughout the text (including statistical abbreviations).

**Response:** Done

* Please ensure that tables and figures, including those in supplementary files, are appropriately referenced in the main text.

**Response:** Done

* Please review your text for claims of novelty or primacy (e.g. 'for the first time' or ‘novel’) and remove this language. 

**Response:** NA

* Please confirm that any use of statistical terms (such as trend or significant) are supported by the data, and if not please remove them. The term trend should be used only when the test for trend has been conducted.

**Response:** NA

* Please provide titles and legends for all figures and tables (including those in Supporting Information files). 

**Response:** Done

* Please define all acronyms used in each figure or table in its corresponding legend.

**Response:** No missing acronyms identified, please advise!

* Please revise for use of patient-centered language. Please note that patient-centered language is constructed with the use of post-modified nouns (e.g. 'patients undergoing surgery’ (or similar) instead of ‘surgery patients’) putting the person first in the sentence structure.

**Response:** Done

* Please confirm that the metadata in the online submission form is updated and accurate.
- The Data Availability Statement (DAS) in the online submission form requires revision. Please ensure to update the statement to include the contact provided in the manuscript file.

**Response:** Done

ABSTRACT
* Please confirm that your abstract complies with our requirements, including providing all the information relevant to this study type [https://journals.plos.org/plosmedicine/s/submission-guidelines#loc-abstract](https://journals.plos.org/plosmedicine/s/submission-guidelines#loc-abstract)  

**Response:** All required information has been added, and the abstract has been revised to comply with the PLOS Medicine guidelines for this study type.

* Please confirm that all numbers presented in the abstract are present and identical to numbers presented in the main manuscript text.

**Response:** Done

* In the abstract, please include the important dependent variables that are adjusted for in the analyses.

**Response:** The key variables included in the adjustment models are already listed in the Abstract (lines 59-60) (“*Analyses were adjusted (adj) for baseline age, sagittal diameter, alcohol consumption, smoking, and serum insulin levels.*”). No additional adjustment variables were used.

* In the last sentence of the Abstract Methods and Findings section, please describe the main limitation(s) of the study's methodology.

**Response:** The following sentence has been added (line 72): *“The main limitation is that cancer was not a predefined study outcome.”*

* We suggest including the original trial registration number in the Abstract Methods.

**Response:** Done.

* l.49, “usual obesity care” – How is usual obesity care defined? Would ‘usual obesity-related care’ be a better description?

**Response:** Thank you for this helpful suggestion. We have clarified what is meant by “usual obesity care” and retained the Editors proposed wording (“obesity-related care”). The revised text in the abstract now states (lines 54-55): *“…matched controls, receiving standard nonsurgical obesity-related care...”*

We have also expanded the description in the section on study design in the manuscript which now states (lines 260-262): *“…controls received the standard nonsurgical obesity-related care provided at their primary health care centers, which was not standardized and ranged from structured lifestyle and behavioral interventions to minimal or no active treatment.”*

* “Moreover, bariatric surgery was associated with a lower overall cancer mortality rate in women (HRadj=0.78 [95% CI:0.61, 1.00]; p=0.050).” – based on the confidence intervals and the p-values, please consider re-phrasing. In the main text, you describe this result as “the association was attenuated”.

**Response:** We thank the editors for highlighting this point and have corrected the phrasing. This sentence now reads (lines 65-66): *“The HRadj for overall cancer mortality rate in women was 0.78 (95% CI [0.61, 1.00]; p=0.050).”*

* Abstract Background: “defined by previously suggested predictors of treatment benefit.” – please provide more detail.

**Response:** The sentence has been modified and now reads (line 47): *“This study examines the link between bariatric surgery and long-term cancer outcomes, focusing on patient subgroups defined by previously suggested predictors of treatment benefit, such as sex and baseline insulin levels.”*

* Abstract Conclusions: We believe you should comment on the male-specific findings here as well.

**Response:** We thank the editors for this suggestion. We have now added a statement summarizing the findings in men (lines 68-69): *“In men, bariatric surgery was not associated with overall cancer incidence or mortality.”*

AUTHOR SUMMARY
* In the author summary, in the final bullet point of 'What Do These Findings Mean?', please clearly state the main limitations of the study in non-technical language.

**Response:** The following text has been added under the final bullet point (lines 196-197): *“The main limitation is that cancer was not a predefined outcome when the study began.”*

METHODS AND RESULTS 
* “age >51 if data were missing” – please add ‘years’ (as a unit) when reporting age.

**Response:** This has been corrected (line 291).

* Thank you for providing your STROBE checklist. Please replace the page numbers with paragraph numbers per section (e.g. "Methods, paragraph 1"), since the page numbers of the final published paper may be different from the page numbers in the current manuscript.

**Response:** Done.

* Table 1: It seems that more women in the surgery group had a prior cancer diagnosis. Is ~1.5 years a considerable age difference? Please verify that you have accurately described the baseline characteristics and their differences.

**Response:** We thank the editors for noting this issue. This text was a remnant from an earlier manuscript version that referenced the now-removed p-values in Table 1. It is correct that the 1.5-year age difference between the groups, while small in absolute terms, could influence comparisons; however, all analyses were adjusted for predefined risk factors, including age.

The text has now been updated to accurately reflect the baseline differences and reads (lines 387-389): *“Women in the surgery group were more often smokers (25.8% vs 19.7%) and had slightly higher prior cancer rates (7.4% vs 5.9%) compared to female control participants. Baseline alcohol intake did not differ between groups (****Table 1****).”*

* “Sensitivity analyses produced similar estimates after excluding 46 women diagnosed within the first three years (HRadj=0.82 [95% CI: 0.70,0.95]; p=0.010) and 190 with prior cancer (HRadj=0.82 [95% CI: 0.70, 0.97]; p=0.017).” – please ensure to include a reference to the relevant table/figure.

**Response:** References to the relevant figures have now been added. These sensitivity analysis results are presented in Supplementary Figure 3 and Supplementary Figure 4.

* “Among female controls, higher baseline insulin levels were associated with higher overall cancer incidence, with rates ranging from 11.6 cases per 1,000-person-years [95% CI: 9.9, 13.7] in the lowest third to 17.8 cases per 1,000-person-years [95% CI: 15.0, 21.2] in the highest third (Figure 3A).” – Please revise for clarity. Instead of describing an association, we suggest simply reporting that higher case numbers were observed in all surgery groups at all insulin levels.

**Response:** Thank you for the helpful suggestion. We have revised the sentence to simply report the observed case numbers and rates across insulin tertiles without implying an association. The revised text now reads (lines 468-471):

*“Among female controls, cancer incidence rates increased across insulin tertiles, ranging from 11.6 cases per 1,000-person-years (95% CI [9.9, 13.7]) in the lowest third to 17.8 cases per 1,000-person-years (95% CI [15.0, 21.2]) in the highest third (****Figure 3A****)”*

* Figure 4: To avoid using the word "fatal," we suggest renaming the graphs. Cancer-related mortality

**Response:** Renamed as suggested.

* In the Methods section, please ensure that you have described how the insulin thresholds were chosen and what constitutes a normal/healthy level, citing relevant literature.

**Response:** We apologize if this was not clear. For interaction analyses, insulin was modeled as a continuous variable using an interaction term in the Cox proportional hazards models. The categorical cut-offs used for descriptive presentation were data-driven (tertiles) and not used for inference. The lowest insulin tertile corresponds to approximately normal insulin levels. To clarify this, we have added the following text to the Methods section (lines 351-355):

*“For descriptive purposes, cancer incidence and mortality were presented across data-driven tertiles of baseline insulin, selected to provide balanced subgroup sizes and to align with prior SOS analyses using quantile-based stratification . No established clinical insulin thresholds exists, however, the lowest insulin third [14, 15] in the SOS cohort corresponds to approximately normal levels [21].”*

------------------------------------------------------------
General Editorial Requests

1) We ask every co-author listed on the manuscript to fill in a contributing author statement. If any of the co-authors have not filled in the statement, we will remind them to do so when the paper is revised. If all statements are not completed in a timely fashion this could hold up the re-review process. Should there be a problem getting one of your co-authors to fill in a statement we will be in contact. YOU MUST NOT ADD OR REMOVE AUTHORS UNLESS YOU HAVE ALERTED THE EDITOR HANDLING THE MANUSCRIPT TO THE CHANGE AND THEY SPECIFICALLY HAVE AGREED TO IT.

**Response:** No authors have been added or removed.

2) Please ensure that the paper adheres to the PLOS Data Availability Policy (see <http://journals.plos.org/plosmedicine/s/data-availability)>, which requires that all data underlying the study's findings be provided in a repository or as Supporting Information. For data residing with a third party, authors are required to provide instructions with contact information for obtaining the data. PLOS journals do not allow statements supported by "data not shown" or "unpublished results." For such statements, authors must provide supporting data or cite public sources that include it.

**Response:** Done

3) Please review your reference list to ensure that it is complete and correct. If you have cited papers that have been retracted, please include the rationale for doing so in the manuscript text, or remove these references and replace them with relevant current references. Any changes to the reference list should be mentioned in the rebuttal letter that accompanies your revised manuscript.

**Response:** Done
